# CAR^+^ extracellular vesicles predict ICANS in patients with B cell lymphomas treated with CD19-directed CAR T cells

**DOI:** 10.1172/JCI173096

**Published:** 2024-06-04

**Authors:** Gianluca Storci, Francesco De Felice, Francesca Ricci, Spartaco Santi, Daria Messelodi, Salvatore Nicola Bertuccio, Noemi Laprovitera, Michele Dicataldo, Lucrezia Rossini, Serena De Matteis, Beatrice Casadei, Francesca Vaglio, Margherita Ursi, Francesco Barbato, Marcello Roberto, Maria Guarino, Gian Maria Asioli, Mario Arpinati, Pietro Cortelli, Enrico Maffini, Enrica Tomassini, Marta Tassoni, Carola Cavallo, Francesco Iannotta, Maria Naddeo, Pier Luigi Tazzari, Elisa Dan, Cinzia Pellegrini, Serafina Guadagnuolo, Matteo Carella, Barbara Sinigaglia, Chiara Pirazzini, Caterina Severi, Paolo Garagnani, Katarzyna Malgorzata Kwiatkowska, Manuela Ferracin, Pier Luigi Zinzani, Massimiliano Bonafè, Francesca Bonifazi

**Affiliations:** 1 IRCCS Azienda Ospedaliero-Universitaria di Bologna, Bologna, Italy.; 2 Dipartimento di Scienze Mediche e Chirurgiche, Università di Bologna, Bologna, Italy.; 3 IRCCS Istituto Ortopedico Rizzoli, Bologna, Italy.; 4 Institute of Molecular Genetics, National Research Council of Italy, Bologna, Italy.; 5 Department of Biomedical and Neuromotor Sciences, Bellaria Hospital, Università di Bologna, Bologna, Italy.; 6 IRCCS Institute of Neurological Sciences of Bologna, Bellaria Hospital, Bologna, Italy.; 7 Laboratory Ramses, Research & Innovation Technology Department, IRCCS Istituto Ortopedico Rizzoli, Bologna, Italy.; 8 Abbelight, Cachan, France.; 9 IRCCS Azienda Ospedaliero–Università di Bologna, Istituto di Ematologia “Seràgnoli,” Bologna, Italy.

**Keywords:** Hematology, Immunology, Immunotherapy, Lymphomas

## Abstract

**BACKGROUND:**

Predicting immune effector cell–associated neurotoxicity syndrome (ICANS) in patients infused with CAR T cells is still a conundrum. This complication, thought to be consequent to CAR T cell activation, arises a few days after infusion, when circulating CAR T cells are scarce and specific CAR T cell–derived biomarkers are lacking.

**METHODS:**

CAR^+^ extracellular vesicle (CAR^+^EV) release was assessed in human CD19.CAR T cells cocultured with CD19^+^ target cells. A prospective cohort of 100 patients with B cell lymphoma infused with approved CD19.CAR T cell products was assessed for plasma CAR^+^EVs as biomarkers of in vivo CD19.CAR T cell activation. Human induced pluripotent stem cell–derived (iPSC-derived) neural cells were used as a model for CAR^+^EV-induced neurotoxicity.

**RESULTS:**

In vitro release of CAR^+^EVs occurs within 1 hour after target engagement. Plasma CAR^+^EVs are detectable 1 hour after infusion. A concentration greater than 132.8 CAR^+^EVs/μL at hour +1 or greater than 224.5 CAR^+^EVs/μL at day +1 predicted ICANS in advance of 4 days, with a sensitivity and a specificity outperforming other ICANS predictors. ENO2^+^ nanoparticles were released by iPSC-derived neural cells upon CAR^+^EV exposure and were increased in plasma of patients with ICANS.

**CONCLUSION:**

Plasma CAR^+^EVs are an immediate signal of CD19.CAR T cell activation, are suitable predictors of neurotoxicity, and may be involved in ICANS pathogenesis.

**TRIAL REGISTRATION:**

NCT04892433, NCT05807789.

**FUNDING:**

Life Science Hub–Advanced Therapies (financed by Health Ministry as part of the National Plan for Complementary Investments to the National Recovery and Resilience Plan [NRRP]: E.3 Innovative health ecosystem for APC fees and immunomonitoring).

## Abstract 2

The release of CAR^+ ^extracellular vesicles by CD19.CAR T cells upon target engagement is a reliable in vivo predictor of immune effector cell–associated neurotoxicity syndrome.

## Graphical abstract

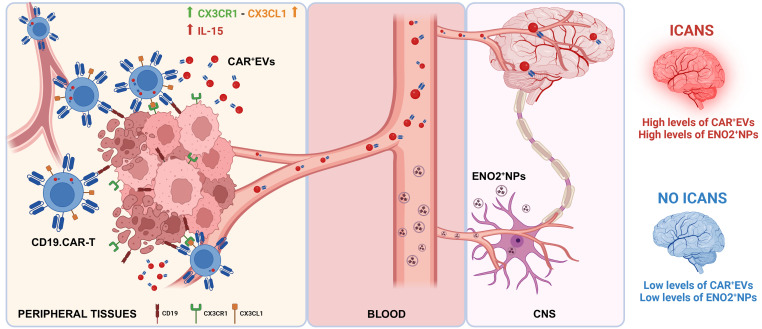

## Introduction

Anti-CD19 chimeric antigen receptor T (CD19.CAR T) cell therapy changed the treatment of B cell malignancies (1). So far, three CD19.CAR T cell products have received approval from regulatory agencies, including the Italian Medicines Agency (AIFA), for the treatment of relapsed/refractory B cell malignancies: axicabtagene ciloleucel (axi-cel) for diffuse large B cell lymphoma (DLBCL), primary mediastinal B cell lymphoma (PMBCL) (2, 3), and follicular lymphoma (FL) (4); tisagenlecleucel (tisa-cel) for DLBCL (5), FL (6), and B cell acute lymphoblastic leukemia (B-ALL) in patients up to 25 years of age (7); and brexucabtagene autoleucel (brexu-cel) for mantle cell lymphoma (MCL) (8) and B-ALL (9). The most common adverse events in CD19.CAR T therapies are the cytokine release syndrome (CRS) and the immune effector cell–associated neurotoxicity syndrome (ICANS) (10). ICANS is clinically characterized by encephalopathy, cognitive impairment, language disturbances, seizures, and, rarely, cerebral edema (11–15). Notably, ICANS has been reported to occur with different incidence depending on the infusion product, specifically 59%–65% for axi-cel (2–4), 21%–40% for tisa-cel (5–7), and 60%–63% for brexu-cel (8, 9). Several factors have been reported to be associated with ICANS: age, performance status, disease burden, disease type, infused CAR T cell dosage, presence and severity of CRS and its shorter time to onset, and presence of preexistent neurological comorbidities (11–17). Although ICANS is rarely fatal (1%–2%), its management requires high-dose steroids and immunosuppressive therapies, which potentially affect CAR T cell activity and therapy efficacy, as well as non-relapse mortality (18). The pathogenesis of ICANS has been associated with endothelial damage, blood-brain barrier disruption, and glial cell injury (19). High C-reactive protein and serum ferritin levels (i.e., baseline inflammatory status), as well as IL-2, IL-3, IL-6, IL-10, IL-15, MCP-1, GM-CSF, and IFN-γ levels measured after CAR T cell infusion, have been reported to be associated with increased risk of ICANS (11, 12, 20). Owing to the lack of tissue specificity, these biochemical parameters can only presumably be related to the functional dynamics of infused CAR T cells. The latter can be assessed by the direct examination of lymph nodes and tumor tissues after CAR T cell infusion, which are, however, not easily accessible in clinical practice. This issue is particularly relevant, even for the demonstration of central nervous system (CNS) involvement during ICANS, given that CAR T cells have been found both in the parenchyma (11) and in the cerebrospinal fluid (12). Plasma/serum extracellular vesicles (EVs) are nano-sized structures released from all cell types, which can mirror the cell-of-origin membrane phenotype (21, 22). Importantly, EVs mediate cell-cell crosstalk and take part in a variety of biological processes, such as immune and inflammatory responses (22). In this regard, it has been previously reported that CAR T cells are capable of releasing EVs that carry the CAR construct (23, 24). Following this observation, we hypothesized that the assessment of CAR T–specific plasma EVs (CAR^+^EVs) could provide information about CAR T cell functioning and trafficking in vivo. We investigated plasma CAR^+^EVs as markers of in vitro CAR T cell activation and as potential plasma predictors of ICANS in CAR T patients. We generated human induced pluripotent stem cell–derived (iPSC-derived) neural cells to model in vitro neurotoxicity by measuring the CAR^+^EV-induced release of Enolase 2–positive (ENO2^+^) nanoparticles (25), which we also searched for in the plasma of patients with ICANS.

## Results

### Patients’ characteristics.

Patients with relapsed/refractory B cell lymphoma (*n* = 100; Table 1) were enrolled in this study. Lymphoma subtypes were as follows: 67 patients (67%) with diffuse large B cell lymphoma (DLBCL and transformed DLBCL), 12 patients (12%) with primary mediastinal B cell lymphoma (PMBCL), 6 patients (6%) with high-grade B cell lymphoma, 1 patient (1%) with gray zone lymphoma, and 14 patients (14%) with mantle cell lymphoma. Twenty-eight patients (28%) received tisa-cel, 58 (58%) received axi-cel, and 14 (14%) received brexu-cel, after a median number of 3 previous lines of treatment (range 1–11). The median age was 60 years (range 19–76), and no patients had CNS disease at the time of CD19.CAR T cell infusion. All patients underwent CAR T cell infusion after standard lymphodepleting chemotherapy with cyclophosphamide (250–500 mg/m^2^) and fludarabine (25–30 mg/m^2^), administered intravenously on days –5, –4, and –3 according to the standard practice. After CAR T cell infusion, 90 (90%) of the 100 patients developed CRS of any grade (Supplemental Table 1; supplemental material available online with this article; https://doi.org/10.1172/JCI173096DS1); 28 patients (28%) developed grade ≥2 CRS, and no patients developed fatal CRS (Table 1). The median time from CAR T cell infusion to CRS onset was 1 day (range 0–11). CRS patients were treated with tocilizumab as first line per standard local procedures. Univariate analyses showed that only CAR T cell product (CD28 costimulated) correlated with CRS occurrence (*P* = 0.045; Supplemental Table 1).

### ICANS after CAR T cell infusion.

Within 30 days after CD19.CAR T cell infusion, 29 (29%) of the 100 patients developed neurotoxicity of any grade. Nine patients (9%) developed grade 1 ICANS, 10 (10%) grade 2, 5 (5%) grade 3, 3 (3%) grade 4, and 2 (2%) grade 5 (fatal neurotoxicity with diffuse cerebral edema) (Table 1). The median time from CAR T cell infusion to the appearance of the first neurological symptom was 5 days (range 3–13). According to previous reports (11–15, 26), all ICANS occurred either after or concomitantly with CRS, with a median interval between the onset of CRS and ICANS of 4 days (range 0–11). Encephalopathy with variable symptoms such as ideomotor slowing, inattention, disorientation, dyscalculia, and confusion was the most frequent finding (27/29, 93%), which was predominantly frontal (17/27, 63%), characterized by motor and specifically verbal and writing perseveration such as palilalia and paligraphia (rewriting of letters, words, and/or sentences), as we previously reported (27). Language disorders (dysarthria and aphasia) were also frequent (20/29, 69%), as well as nonspecific ICANS symptoms, such as postural tremors (24/29, 83%) and headache (10/29, 34%). In 3 cases (1 patient with grade 4 ICANS and 2 patients with grade 5 ICANS), the clinical onset was characterized by severe headache and vomiting related to intracranial hypertension, followed by a rapid deterioration to a coma within a few hours. Contrast brain MRI was performed in 25 of 29 patients with ICANS (86%); no abnormalities were found in 18 of the 25 patients (72%), whereas in the remaining patients (28%) new abnormalities were found, such as focal cerebral edema and leptomeningeal enhancement (*n* = 3), focal intraparenchymal and subarachnoid hemorrhage (*n* = 1), and diffuse cerebral edema (*n* = 3). Electroencephalography documented predominantly diffuse slowing of electrical activity in almost all (93%) patients with ICANS, and epileptic abnormalities (without clinical seizures) were observed in 4 patients (14%). Patients with ICANS were treated with high-dose steroids as first line per standard local procedures; siltuximab and anakinra were added for grade ≥3. All patients resolved ICANS except for 2 patients with grade 5. Univariate analyses showed the association of the following variables with ICANS occurrence: Eastern Cooperative Oncology Group performance scale (ECOG) (≥1), disease histotype (PMBCL), disease status at infusion (progressive disease), bridging therapy (immune checkpoint inhibitors), and grade ≥2 CRS (Table 1). Multivariate analysis confirmed ECOG ≥1 (OR = 4.42, 95% CI: 1.10–19.19, *P* = 0.038), PMBCL (OR = 51.19, 95% CI: 6.93–1,123.76, *P* = 0.001), progressive disease status at infusion (OR = 10.23, 95% CI: 1.84–192.19, *P* = 0.03), and grade ≥2 CRS (OR = 4.55, 95% CI: 1.49–14.58, *P* = 0.008) as clinical risk factors for ICANS.

### CAR^+^T cells and CAR_DNA expansion kinetics.

**Figure 1:**
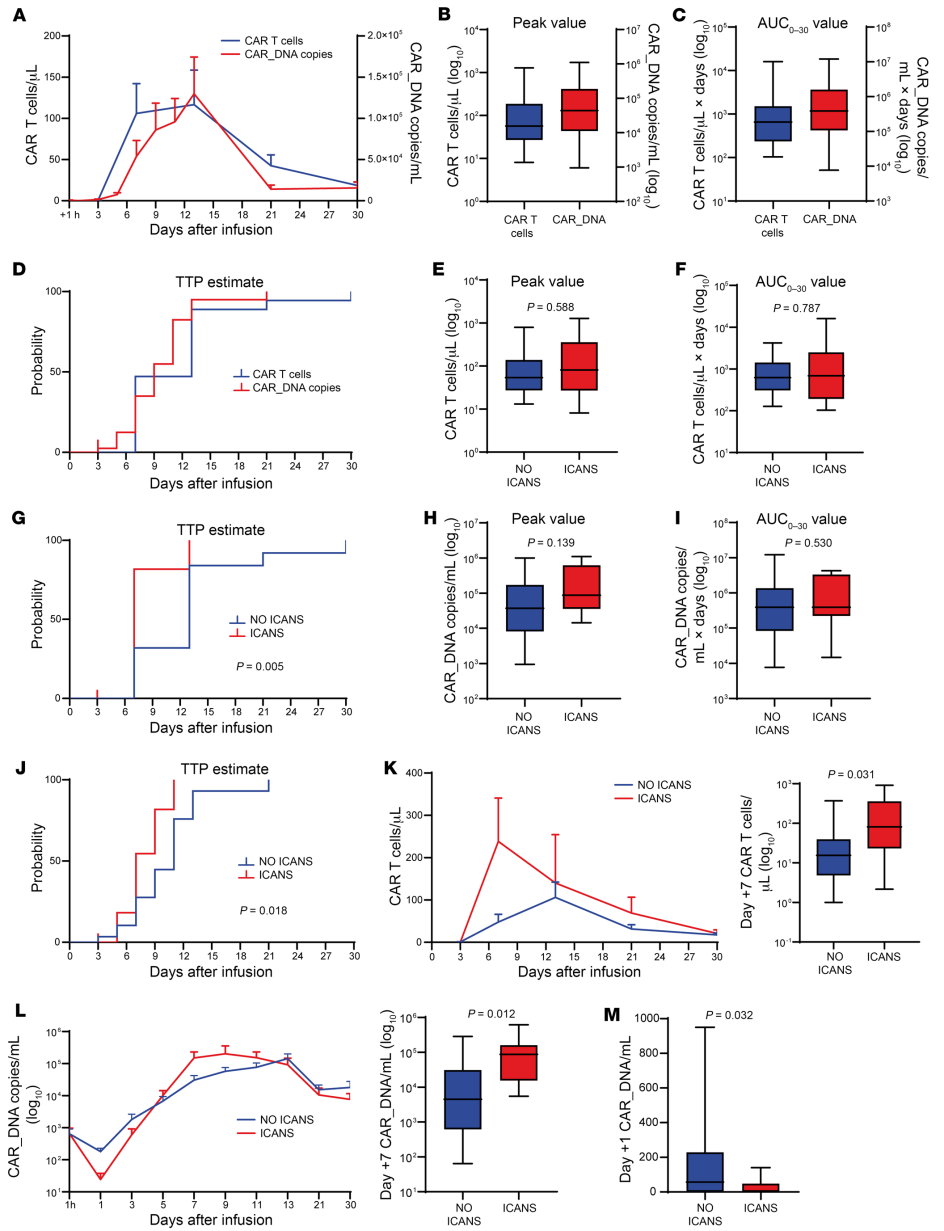
Kinetics of peripheral blood CAR^+^T cells and CAR_DNA copies in CAR T cell–infused patients. (**A**) Peripheral blood kinetics of CAR^+^T cells (*n* = 37) and CAR_DNA copies (*n* = 41), assessed by MFC and ddPCR, respectively (mean ± SEM). (**B**) Expansion peak values of CAR^+^T cells (*n* = 36) and CAR_DNA copies (*n* = 40). (**C**) AUC_0–30_ value estimates for CAR^+^T cells (*n* = 36) and CAR_DNA copies (*n* = 40). (**D**) Kaplan-Meier (KM) estimate of time to peak (TTP) for CAR^+^T cells (*n* = 37) and CAR_DNA copies (*n* = 41). (**E**) CAR^+^T cell expansion peak values in NO ICANS (*n* = 25) versus ICANS (*n* = 11); Mann-Whitney (MW) test. (**F**) CAR^+^T cell AUC_0–30_ value estimate in NO ICANS (*n* = 25) versus ICANS (*n* = 11); MW test. (**G**) KM estimate of CAR^+^T cell TTP in NO ICANS (*n* = 25) versus ICANS (*n* = 12); log-rank test. (**H**) Expansion peak values of CAR_DNA copies in NO ICANS (*n* = 29) versus ICANS (*n* = 11); MW test. (**I**) AUC_0–30_ value estimates for CAR_DNA copies in NO ICANS (*n* = 29) versus ICANS (*n* = 11); MW test. (**J**) KM estimate of TTP for CAR_DNA copies in NO ICANS (*n* = 29) versus ICANS (*n* = 12); log-rank test. (**K**) Peripheral blood kinetics of CAR^+^T cells in NO ICANS (*n* = 25) versus ICANS (*n* = 12), and day +7 CAR^+^T cells in NO ICANS (*n* = 25) versus ICANS (*n* = 11); MW test. (**L**) Kinetics of CAR_DNA copies (mean ± SEM) in NO ICANS (*n* = 29) versus ICANS (*n* = 12), and day +7 CAR_DNA copies in NO ICANS (*n* = 29) versus ICANS (*n* = 7); MW test. (**M**) Day +1 CAR_DNA copies in NO ICANS (*n* = 29) versus ICANS (*n* = 12); MW test. Unless otherwise indicated, data are presented as boxes and whiskers; boxes show median and interquartile range (IQR), and whiskers represent minimum and maximum values.

The 30-day kinetics of CAR^+^T cells expressed as mean cell number per microliter of blood (assessed by multi-flow cytometry [MFC]) and mean CAR_DNA copies per milliliter of blood (assessed by droplet digital PCR [ddPCR]) is shown in Figure 1A. The median expansion peak values of CAR^+^T cells and CAR_DNA copies were 57 (range 8–1,279) cells/μL and 44,167 (range 950–1,105,000) copies/mL (Figure 1B). The median values of areas under the curve over 30 days after infusion (AUC_0–30_) were 654 (range 104–15,997) CAR^+^T cells/μL × days and 388,881 (range 7,673–12,096,513) CAR_DNA copies/mL × days (Figure 1C). The median time to peak (TTP) was 13 (range 7–30) days and 9 (range 3–21) days for CAR^+^T cells and CAR_DNA copies, respectively (Figure 1D). Patients developing ICANS showed no significant differences in median CAR^+^T cell expansion peak and AUC_0–30_ values compared with patients who did not develop ICANS (NO ICANS patients): 81 versus 54 CAR^+^T cells/μL (Figure 1E), 688 versus 620 CAR^+^T cells/μL × days (Figure 1F). In contrast, a shorter CAR^+^T cells/μL median TTP was observed in ICANS (7, range 7–13 days) compared with NO ICANS (13, range 7–30 days) patients (*P* = 0.005; Figure 1G). Consistently, ddPCR confirmed similar results of MFC analyses: 87,413 versus 37,000 median CAR_DNA copies/mL at expansion peak (Figure 1H) and 388,530 versus 389,231 median CAR_DNA copies/mL×days (AUC_0–30_) in ICANS versus NO ICANS patients (Figure 1I). Shorter CAR_DNA copies/mL median TTP was finally observed in ICANS (7, range 5–11 days) versus NO ICANS (11, range 3–21 days) patients (Figure 1J). The comparison of day +7 CAR^+^T cells and CAR_DNA copies revealed increased levels in patients developing ICANS versus NO ICANS ones (81 vs. 15 CAR^+^T cells/μL, Figure 1K; 87,413 vs. 4,500 CAR_DNA copies/mL, Figure 1L). More importantly, we observed significantly lower levels of day +1 CAR_DNA copies/mL in patients who would later develop ICANS (0 vs. 57 CAR_DNA copies/mL; Figure 1M). Since CAR_DNA copies/mL detected at 1 hour after infusion were not different between ICANS and NO ICANS patients (Figure 1L), we hypothesized that the drop in the blood CAR_DNA copies/mL represents an early homing of CD19.CAR T cells into the tumor and/or peripheral tissues (CNS included). This phenomenon is likely to occur to an increased extent in patients who will develop neurotoxicity in the subsequent days. Hence, the accelerated CD19.CAR T cell kinetics (a shorter TTP) in patients with ICANS is expected to be consequent to an earlier CD19.CAR T cell homing and activation, marked by the drop of day +1 CAR_DNA copies.

### IL-15 and CX3CL1: early players in CD19.CAR T cell homing and activation.

**Figure 2:**
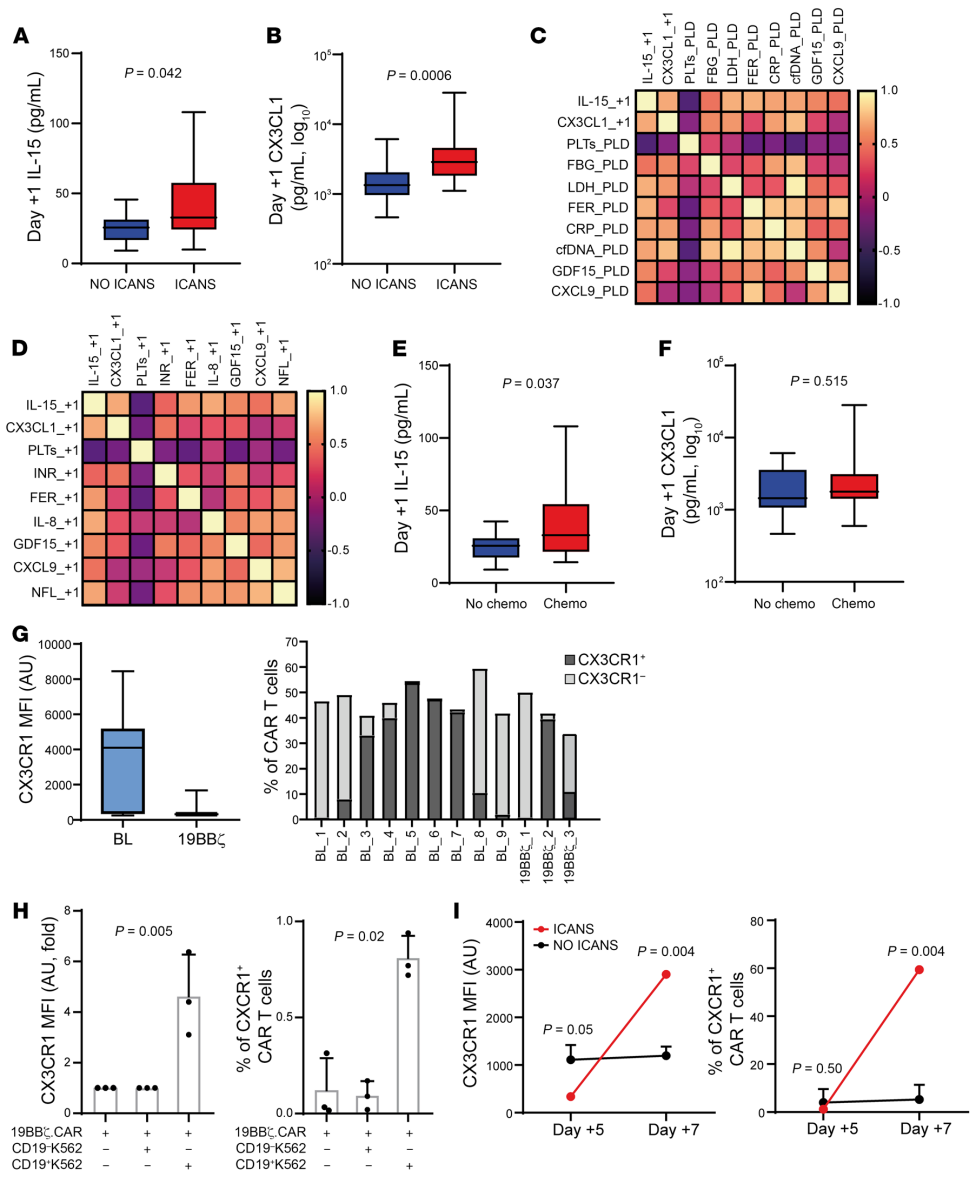
IL-15 and CX3CL1/CX3CR1 interplay in ICANS and NO ICANS patients. (**A**) Day +1 IL-15 plasma levels in NO ICANS (*n* = 21) versus ICANS (*n* = 11); MW test. (**B**) Day +1 CX3CL1 plasma levels in NO ICANS (*n* = 36) versus ICANS (*n* = 17); MW test. (**C** and **D**) Heatmaps (Pearson’s *r* coefficients) of day +1 IL-15 and CX3CL1 plasma levels and biochemical profile at pre-lymphodepletion (_PLD) (**C**) and day +1 (_+1) (**D**). (**E**) Day +1 IL-15 plasma levels in patients treated with chemotherapy as bridging therapy (Chemo, *n* = 13) versus others (No chemo, *n* = 19); MW test. (**F**) Day +1 plasma CX3CL1 in No chemo (*n* = 36) versus Chemo (*n* = 17); MW test. (**G**–**I**) CX3CR1 mean fluorescence intensity (MFI) and percentage of CX3CR1^+^ cells in bag leftover (BL, *n* = 9) and 19BBζ.CAR T cells (*n* = 3) (**G**), 19BBζ.CAR T cells cocultured with CD19^+^ or CD19^−^K562 cells (*n* = 3) for 1 hour (logFC *t* test and paired *t* test; mean ± SD) (**H**), and day +5 and day +7 peripheral blood CD19.CAR T cells in ICANS (*n* = 1) and NO ICANS (*n* = 3, 1-sample *t* test; mean ± SD) (**I**). Unless otherwise indicated, data are presented as boxes and whiskers; boxes show median and IQR, and whiskers represent minimum and maximum values.

Based on the observations above, we focused our attention on IL-15, a cytokine already reported to be associated with ICANS (28, 29), and on CX3CL1, named fractalkine, a chemokine that plays a major role in the tissue homing of CX3CR1^+^CD8^+^ lymphocytes (30). We therefore measured plasma IL-15 and CX3CL1 levels at day +1 after infusion, finding significantly higher median levels of both cytokines in patients with ICANS: 26 versus 33 pg/mL (*P* = 0.042) for IL-15 and 1,344 versus 2,869 pg/mL (*P* = 0.0006) for CX3CL1 (Figure 2, A and B). As expected, IL-15 and CX3CL1 levels measured at day +1 were correlated with each other (*r* = 0.653, *P* = 0.00007) and with variables known to be associated with inflammatory status and/or tumor burden, namely low platelet count (PLT) and high levels of fibrinogen (FBG), lactate dehydrogenase (LDH), ferritin (FER), C-reactive protein (CRP), cell-free DNA (cfDNA), growth differentiation factor 15 (GDF15), CXCL9, and neurofilaments (NFL), measured both at pre-lymphodepletion and at day +1 (Figure 2, C and D). Notably, these biochemical parameters have been extensively reported as risk factors for the development of CAR T–related neurotoxicity and regarded as predictors of poor outcome (20, 31). IL-15, a trigger for the activation of CAR T cells, is also produced upon prior chemotherapies (bridging and/or lymphodepletion) (28). Here, we found higher levels of day +1 IL-15 but not CX3CL1 (*P* = 0.515) in patients who underwent chemo-based bridging therapy, compared with those receiving no chemo-based therapies: 33 versus 26 pg/mL (*P* = 0.037) (Figure 2, E and F). With the aim of elucidating the role of CX3CL1 in CD19.CAR T cell biology, we investigated the expression of its receptor (CX3CR1) on the surface of CD19.CAR T cells. We found highly heterogeneous levels of CX3CR1 expression in 9 bag leftovers (BLs) and 19BBζ.CAR T cells generated from 3 healthy donors (Figure 2G). CX3CR1 expression increased readily after 1 hour of cocultures of 19BBζ.CAR T cells with target CD19^+^K562 cells compared with CD19^−^K562 cells (Figure 2H). In keeping with these results, CX3CR1 expression on CAR^+^T cells was upregulated at day +7 in a patient developing ICANS, but not in three NO ICANS ones (Figure 2I). Since CX3CL1/CXCR1 interplay acts as neurotropic axis, activated CD19.CAR T cells may be more likely to be attracted into the CNS, as well as into CX3CL1-releasing tissues (32, 33). Hence, these data suggest that the CX3CR1/CX3CL1 axis may take part in ICANS pathogenesis, as a likely player in CD19.CAR T cell tissue homing.

### CAR^+^EVs release in vitro upon CD19.CAR T cell activation.

**Figure 3:**
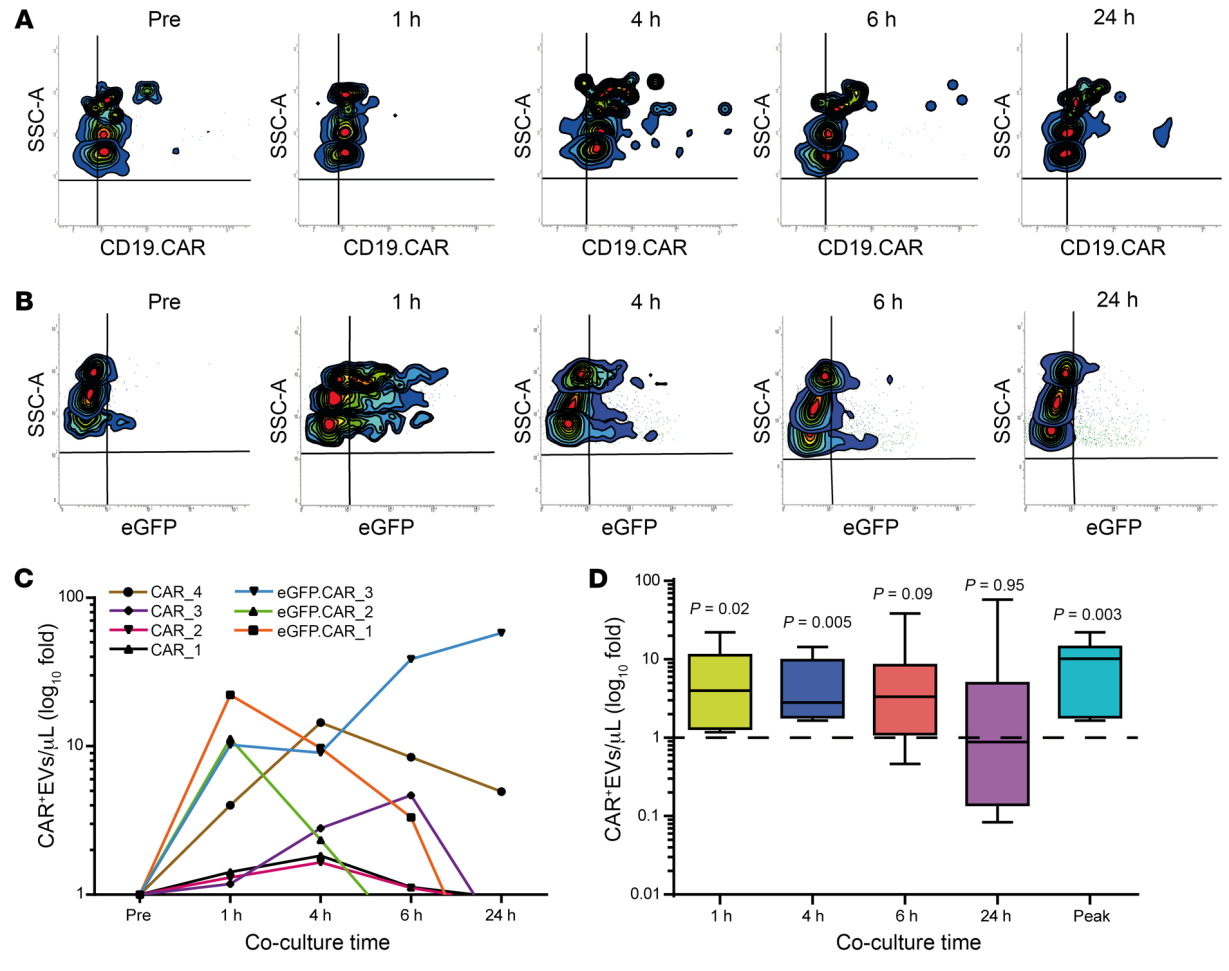
MFC analysis of CAR^+^EVs in the supernatants of 19BBζ.CAR T or 19BBζ.eGFP.CAR T cells cocultured with CD19^+^ target cells. MFC analysis of CAR^+^EVs released in vitro in coculture supernatants of 19BBζ.CAR T or 19BBζ.eGFP.CAR T cells with CD19^+^ target cells. (**A** and **B**) Representative scatter plots. (**C**) CAR^+^EV kinetics for each coculture (19BBζ.CAR T cells, *n* = 4; 19BBζ.eGFP.CAR T cells, *n* = 3). (**D**) CAR^+^EVs in 19BBζ.CAR T and 19BBζ.eGFP.CAR T cell cocultures (*n* = 7) at different time points and at peak level (logFC *t* test; *P* values ≤ 0.02 were considered significant according to Benjamini-Hochberg correction for multiple comparisons). Data are presented as boxes and whiskers; boxes show median and IQR, and whiskers represent minimum and maximum values.

**Figure 4:**
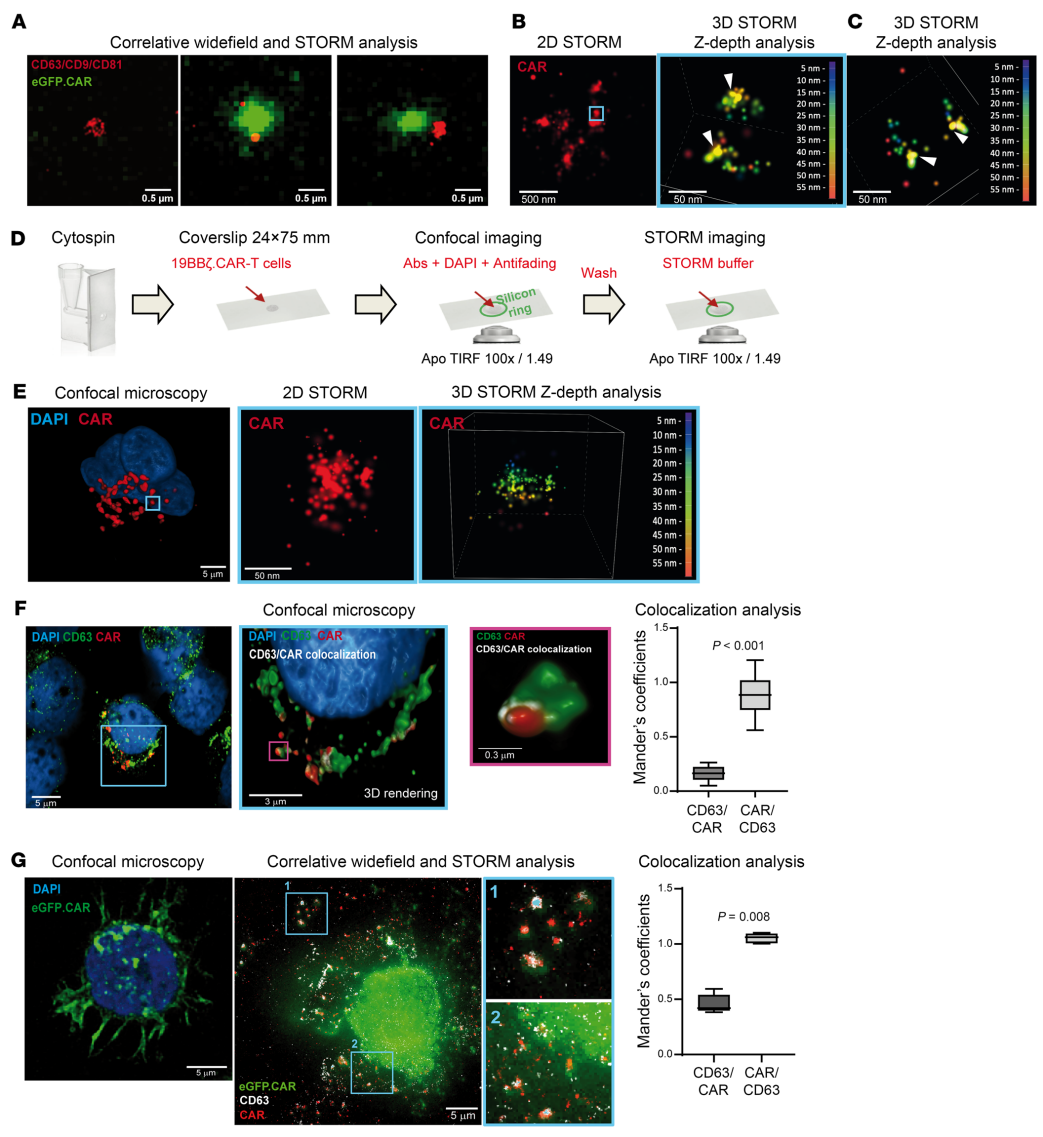
Combined confocal/STORM analysis of 19BBζ.CAR T and 19BBζ.eGFP.CAR T cells and CAR^+^EVs. (**A**) Analysis of 19BBζ.eGFP.CAR T cell coculture supernatants (24 hours): 19BBζ.eGFP.CAR protein (eGFP.CAR; green, wide-field signal) and CD63/CD9/CD81 pool (red, STORM signal). Scale bars: 500 nm (*n* = 6). (**B**) 19BBζ.CAR T cell coculture supernatants (24 hours): 2D-STORM analysis of anti-CD19.CAR Ab–mediated pull-down CAR^+^EVs (CAR; red; scale bar: 500 nm); 3D-STORM analysis of a single CAR^+^EV (colors represent *Z*-depth; scale bar: 50 nm). White arrowheads highlight CD19.CAR antigen clusters on the EV (*n* = 17). (**C**) 3D-STORM analysis (colors represent *Z*-depth; scale bar: 50 nm) of CAR T cell supernatants purified by size-exclusion chromatography. White arrowheads highlight CD19.CAR antigen clusters on the EV (*n* = 3). (**D**) Schematic representation of the combined confocal/STORM analysis workflow. (**E**) Combined confocal/STORM analysis with 3D rendering of a 19BBζ.CAR T cell (CAR, red; DAPI, blue); detail of a CAR^+^ intracellular vesicle observed in 2D- and 3D-STORM (blue box, magnification ×13) (*n* = 3). Scale bars: 5 μm and 50 nm. (**F**) Confocal microscopy imaging of a 19BBζ.CAR T cell (CAR, red; CD63, green; DAPI, blue). Colocalization details (blue box, ×3 magnification; purple box, ×22 magnification) measured by Manders’ overlap coefficients (CD63 over CAR, and CAR over CD63, *n* = 13, MW test) (*n* = 7). Scale bars: 5 μm, 3 μm, and 0.3 μm. (**G**) Combined microscopy of 19BBζ.eGFP.CAR T cells: confocal imaging of eGFP.CAR (green) and DAPI (blue), and correlative wide-field (eGFP.CAR, green)/STORM analysis (CD63, white, and CAR, red). Colocalization details (blue boxes, magnification ×2) measured by Manders’ overlap coefficients (CD63 over CAR, and CAR over CD63, *n* = 5, MW test) (*n* = 2). Scale bars: 5 μm. Data are presented as boxes and whiskers; boxes show median and IQR, and whiskers represent minimum and maximum values.

The data presented above suggest that ICANS may be associated with early CD19.CAR T cell homing and activation. Since in our clinical practice lymphoma specimens of patients treated with CD19.CAR T cells are almost unavailable, we sought a plasma readout of CAR T cell activity upon target engagement. Thus, we tested whether CD19.CAR T cells can release extracellular vesicles (EVs) that carry the chimeric antigen receptor (CAR^+^EVs), on the assumption that such CAR^+^EVs can become detectable in biofluids and be taken as biomarkers of CAR T cell activation. We cultured 19BBζ.CAR T (*n* = 4) and 19BBζ.eGFP.CAR T (*n* = 3) cells in the absence or presence of CD19^+^ target cells (Supplemental Figure 1, A–C, and Supplemental Videos 1 and 2). CAR^+^EV release was assessed by MFC, either via direct eGFP detection or by CD19.CAR protein–specific immunostaining (Figure 3, A and B, and Supplemental Figure 1D). A substantial increase in CAR^+^EV concentration in supernatants of both 19BBζ.CAR T and 19BBζ.eGFP.CAR T cells cocultured with CD19^+^ target cells was invariably observed, within a time frame from 1 to 4 hours (Figure 3, C and D). Combined wide-field/stochastic optical reconstruction microscopy (STORM) analysis on supernatants of 19BBζ.eGFP.CAR T and target cell cocultures confirmed the presence of putative eGFP.CAR^+^EVs upon their capture onto slides coated with anti-tetraspanin (CD63, CD9, CD81) antibodies (Abs) (Figure 4A). Similarly, 2D-/3D-STORM analysis of 19BBζ.CAR T supernatants captured onto poly-lysine–coated slides confirmed the presence of CD19.CAR protein on EVs (Figure 4, B and C). Combined confocal and STORM analysis (Figure 4D) on 19BBζ.CAR T cells revealed a massive presence of CAR^+^ vesicles in the cytoplasm (Figure 4E and Supplemental Figure 1B). In both 19BBζ.CAR T and 19BBζ.eGFP.CAR T cells, most of the CD19.CAR protein was colocalized with CD63 (Figure 4, F and G). Notably, a similar cytoplasmic localization was observed for perforin (PRF1; Supplemental Figure 2, A and B). These data suggest that the CD19.CAR protein, mostly harbored in CD63^+^ cytoplasmic vesicles, is massively released in the supernatant as CAR^+^EVs, mainly upon target engagement.

### CAR^+^EVs are detectable in the plasma of CD19.CAR T cell–infused patients.

**Figure 5:**
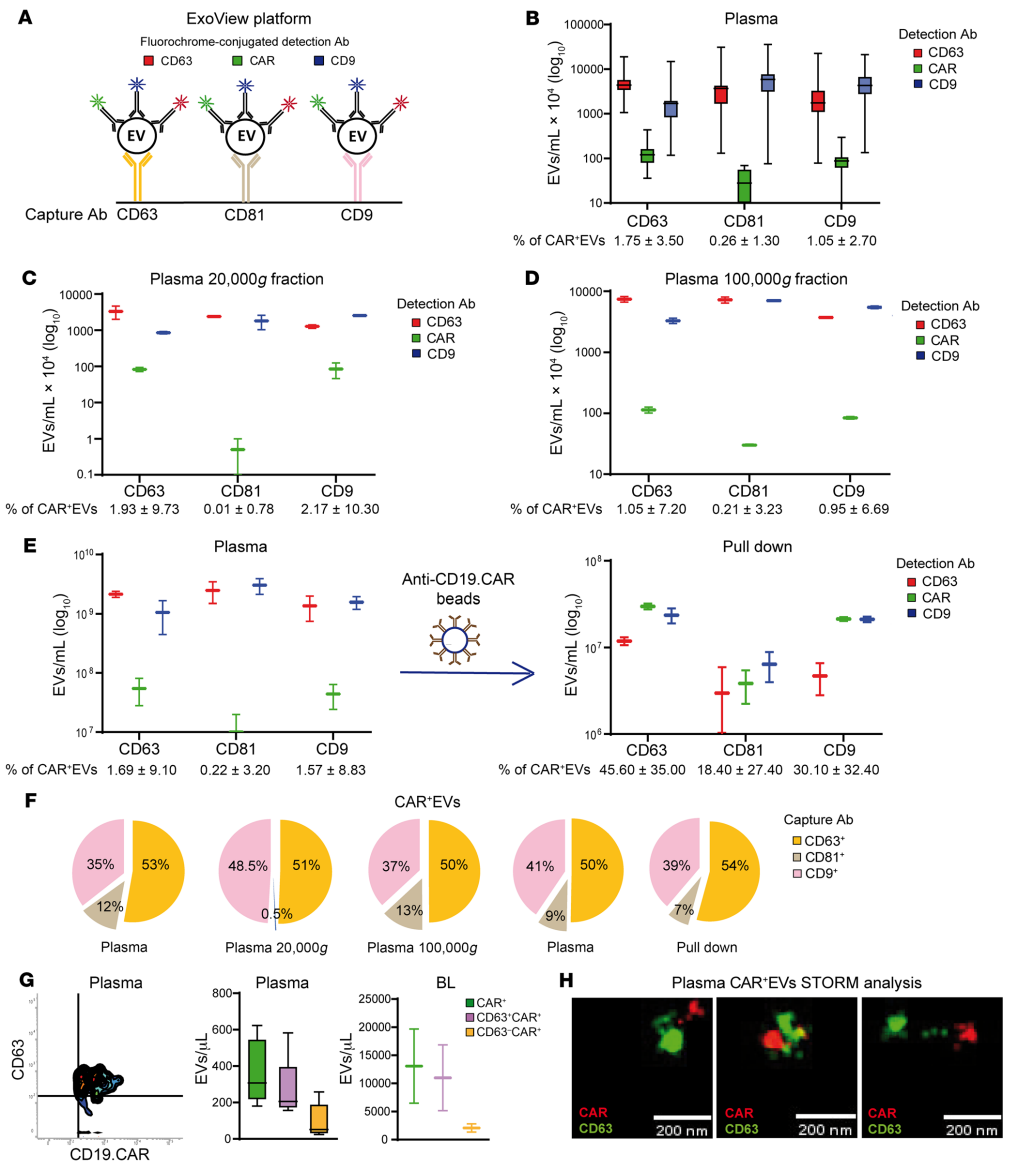
Plasma CAR^+^EV phenotype by ExoView, MFC, and STORM analysis. (**A**) Schematic representation of ExoView platform analysis: CD63-, CD9-, or CD81-immunocaptured CAR^+^EVs are triple-stained by PE–anti-CD19.CAR, Alexa Fluor 647–anti-CD63, and Alexa Fluor 488–anti-CD9 Abs. (**B**–**D**) ExoView analysis of whole (*n* = 14; **B**) (see also Supplemental Figure 5A), 20,000*g* plasma fraction (*n* = 2; **C**), and 100,000*g* plasma fraction (*n* = 2; **D**). (**E**) Anti-CD19.CAR Ab–mediated pull-down of plasma CAR^+^EVs (*n* = 2). (**F**) Pie charts represent the tetraspanin profile of CAR^+^EVs analyzed in **B**–**E**, as ratio of CD63^+^, CD81^+^, or CD9^+^ CAR^+^EVs to total CAR^+^EVs. (**G**) MFC analysis of plasma (*n* = 5) and BL (*n* = 2) CAR^+^EVs. (**H**) Plasma CAR^+^EV STORM analysis. Double-positive CAR^+^CD63^+^ EVs are shown (*n* = 6). Scale bars: 200 nm. Data are presented as boxes and whiskers; boxes show median and IQR, and whiskers represent minimum and maximum values.

Prompted by the above results, we analyzed the presence of CAR^+^EVs in the day +1 plasma of 14 CAR T patients by ExoView. This platform is specifically designed for the immunocapturing of plasma EVs onto CHIPs coated with Abs against established EVs/exosomal markers (CD63, CD9, CD81) assessed by 3-color immunophenotyping (Figure 5A). CAR^+^EVs were 1.75% of CD63-immunocaptured EVs, 0.26% of CD81-immunocaptured EVs, and 1.05% of CD9-immunocaptured EVs (Figure 5B). Moreover, CAR^+^CD9^+^CD63^+^EVs were 8.74% of CD63-immunocaptured CAR^+^EVs, 13.00% of CD9-immunocaptured CAR^+^EVs, and 30.71% of CD81-immunocaptured CAR^+^EVs (Supplemental Figure 3A). The exosome-like profile of CAR^+^EVs was confirmed in both the 20,000*g* (large EVs) and 100,000*g* (small EVs) ultracentrifugation plasma fractions (Figure 5, C and D). Size-exclusion chromatography–purified EVs confirmed the marker profile of the previous analyses even when chimeric CD19 protein was used to detect CAR^+^EVs (Supplemental Figure 3B). The data reported above show that most of the CAR^+^EVs carried CD63 and CD9 exosomal markers, a phenotype that also characterized CAR^+^EVs from BLs (Supplemental Figure 3C). Purification with anti-CD19.CAR–conjugated latex beads yielded an enrichment in CD63^+^CAR^+^EVs (27-fold), CD9^+^CAR^+^EVs (19.2-fold), and CD81^+^CAR^+^EVs (83-fold) (Figure 5E). All the data described above indicate that plasma CAR^+^EVs carry exosomal markers (34) in the following proportions: CD63 around 50%, CD9 around 40%, CD81 less than 12% (Figure 5F). CAR^+^EVs were also measured by MFC, which showed that a large proportion of plasma and BL CAR^+^EVs carried the exosomal marker CD63 (Figure 5G). In keeping with this finding, STORM analysis of plasma EVs, captured onto anti-tetraspanin Ab–coated slides, confirmed the presence of plasma CD63^+^CAR^+^EVs (Figure 5H). Notably, highly clustered CD19.CAR protein was observed on EVs (see also Figure 4, A and B). By ExoView and MFC analysis, we observed that CAR^+^EVs carried PRF1 protein (Supplemental Figure 3, D–F) (35). CAR^+^EVs were almost devoid of the endosomal CD107a/LAMP1 protein but contained a substantial amount of wheat germ agglutinin–binding membrane surface glycoproteins, thus recalling the recently reported supramolecular complexes that are released by cytotoxic T cells upon activation (Supplemental Figure 3, G and H) (36). As previously reported (36), we confirmed that most CAR^+^EVs lack CD45 (Supplemental Figure 3I). Nanosight analysis of FACS-sorted and CD19.CAR pull-down–isolated CAR^+^EVs showed their enrichment in the nano-EV (30–149 nm) and small EV (150–500 nm) compartments (Supplemental Figure 4, A and B). Interestingly, a wide proportion of plasma CD63^+^CAR^+^EVs carried the CD3ζ-associated kinase ZAP70 and the T cell marker CD8β (Supplemental Figure 4C). Similarly, a substantial proportion of sorted CAR^+^EVs carried FAS ligand and the activated T cell tetraspanin CD151 (Supplemental Figure 4, D–F) (37). Finally, MFC analysis of FACS-sorted and CD19.CAR pull-down–isolated CAR^+^EVs revealed the presence of both CD4^+^CAR^+^EVs and CD8^+^CAR^+^EVs, mirroring the cellular CD4^+^/CD8^+^ ratio of the cognate CD19.CAR T cell compartment (Supplemental Figure 5, A–C). Overall, our data show that CAR^+^EVs carry exosomal and T cell markers and can be measured in the plasma of CAR T cell–infused patients.

### CAR^+^EVs: early markers of ICANS in CD19.CAR T cell–infused patients.

**Figure 6:**
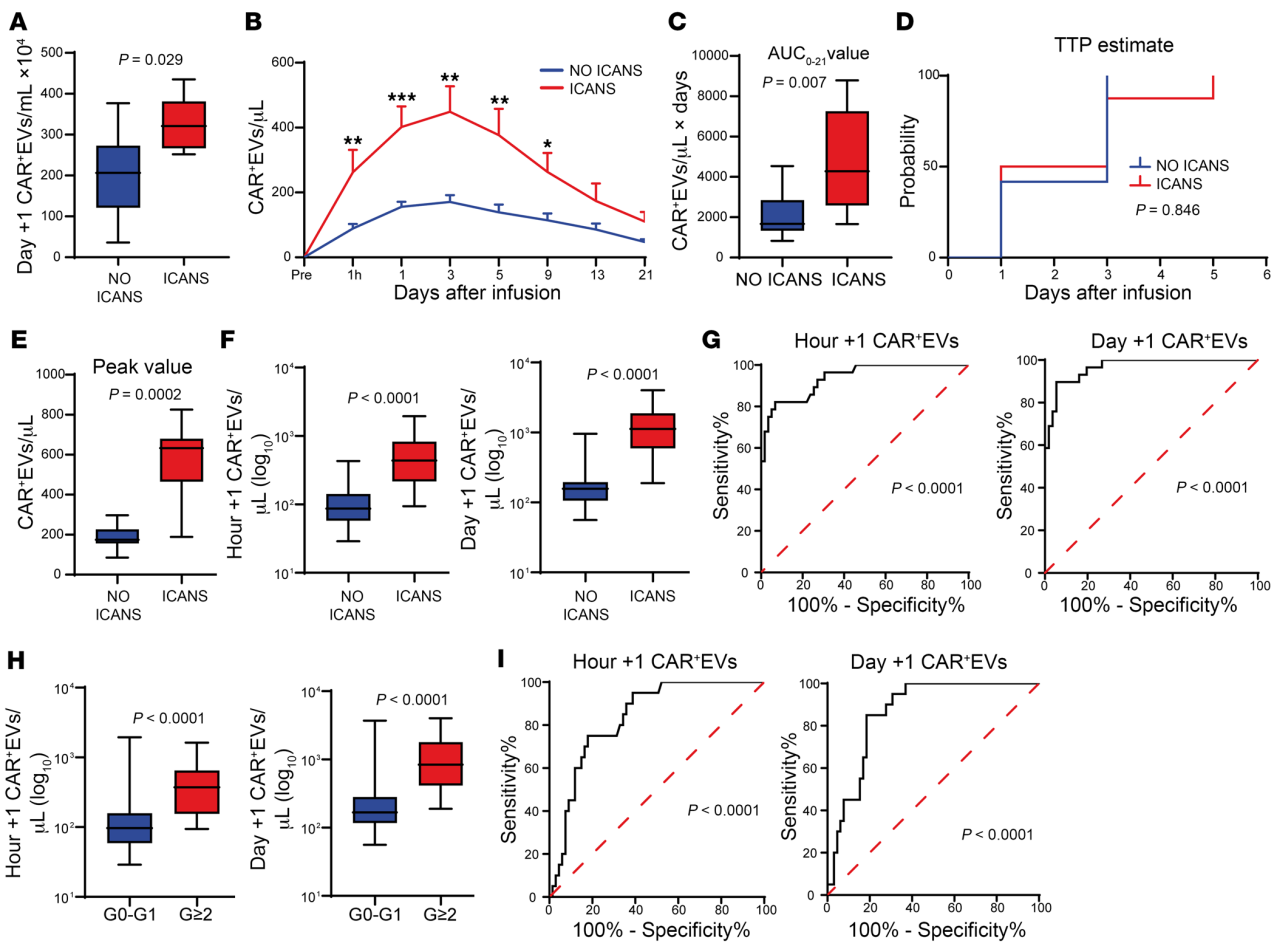
Plasma CAR^+^EVs: early markers of ICANS. (**A**) Day +1 plasma CAR^+^EVs measured by ExoView in NO ICANS (*n* = 8) versus ICANS (*n* = 6); MW test. (**B**–**E**) Plasma CAR^+^EVs assessed by MFC in NO ICANS (*n* = 12) versus ICANS (*n* = 8): (**B**) Twenty-one-day kinetics (mean ± SEM; *P < 0.05, **P < 0.01, ***P < 0.001, multiple MW test). (**C**) AUC_0–21_ estimate value; MW test. (**D**) KM estimate of TTP; log-rank test. (**E**) Peak value; MW test. (**F** and **G**) MFC analysis of hour +1 plasma CAR^+^EVs in NO ICANS (*n* = 59) versus ICANS (*n* = 28) and day +1 plasma CAR^+^EVs in NO ICANS (*n* = 56) versus ICANS (*n* = 29), MW test (**F**); and respective ROC curve analysis (**G**). (**H** and **I**) MFC analysis of hour +1 plasma CAR^+^EVs in grade 0 (G0) to G1 ICANS (*n* = 67) versus G≥2 ICANS (*n* = 20) and day +1 plasma CAR^+^EVs in G0–G1 ICANS (*n* = 65) versus G≥2 ICANS (*n* = 20), MW test (**H**); and respective ROC curve analysis (**I**). Unless otherwise indicated, data are presented as boxes and whiskers; boxes show median and IQR, and whiskers represent minimum and maximum values.

The observations reported above allowed us to investigate the kinetics of plasma CAR^+^EVs and its correlation with ICANS onset. The preliminary analysis of 14 plasma samples at day +1 by ExoView showed that the median number of CAR^+^EVs/μL was higher in the plasma of patients who later developed ICANS compared with NO ICANS ones (321 vs. 207, *P* = 0.029; Figure 6A). As a further preliminary test, we investigated CAR^+^EV kinetics in 20 patients (8 ICANS and 12 NO ICANS) by MFC (Figure 6B). Higher CAR^+^EV AUC_0–21_ median values were found in ICANS compared with NO ICANS patients (4,276 vs. 1,672, *P* = 0.007; Figure 6C); CAR^+^EV TTP was not significantly different (*P* = 0.846; Figure 6D). The CAR^+^EVs/μL median peak value was higher in ICANS compared with NO ICANS patients (633 vs. 175, *P* = 0.0002; Figure 6E). Overall, CAR^+^EV peak anticipated by approximately 7 days the CD19.CAR T cell peaks by both ddPCR and MFC (see Figure 1). As shown above (Figure 6B), the comparison of CAR^+^EVs kinetics in ICANS and NO ICANS patients showed a significant difference at the earlier (e.g., hour +1, day +1) time points. Prompted by this compelling data, we analyzed these time points (hour +1 and day +1) by MFC in all the available plasma samples (*n* = 87). The data confirmed that patients with ICANS had higher plasma CAR^+^EVs/μL median levels as early as hour +1 compared with NO ICANS ones (439 vs. 87, *P* < 0.0001), as well as at day +1 (1,123 vs. 158, *P* < 0.0001) (Figure 6F). Accordingly, receiver operating characteristic (ROC) analysis showed that, above 132.8 CAR^+^EVs/μL at hour +1, the onset of ICANS was predicted with a sensitivity of 89.29% and specificity of 74.58% (*P* < 0.0001), as well as that, above 224.5 CAR^+^EVs/μL at day +1, the onset of ICANS was predicted with sensitivity of 96.55% and specificity of 80.36% (*P* < 0.0001) (Figure 6G). Finally, with the aim of testing the independent predictive value of CAR^+^EVs, we set up 2 multivariate analyses adding CAR^+^EVs (either hour +1 or day +1) to the clinical variables already found to be significantly associated with ICANS development (see Table 1). The number of CAR^+^EVs/μL at hour +1 and day +1 was significantly associated with ICANS (hour +1: OR = 1.02, 95% CI: 1.01–1.03, *P* = 0.00009; day +1: OR = 1.01, 95% CI: 1.00–1.01, *P* = 0.00008) in multiple logistic regression analysis; ECOG ≥1 retained its significance (hour +1 CAR^+^EVs/μL: OR = 11.67, 95% CI: 1.71–87.62, *P* = 0.012; day +1 CAR^+^EVs/μL: OR = 11.36, 95% CI: 1.70–89.63, *P* = 0.013). In contrast, disease histotype (PMBCL), disease status at infusion (progressive disease), bridging therapy (immune checkpoint inhibitors), and grade ≥2 CRS lost their significant association with ICANS in multivariate analysis. Notably, close correlations were observed between ICANS grade and hour +1 CAR^+^EVs/μL (*r* = 0.669, *P* = 1.47 × 10^–12^) and day +1 CAR^+^EVs/μL (*r* = 0.725, *P* = 4.46 × 10^–15^). When patients were grouped according to ICANS severity, i.e., grade 0–1 versus grade ≥2, CAR^+^EVs/μL levels showed a median value of 97 versus 374 (*P* < 0.0001) at hour +1, and a median value of 167 versus 840 (*P* < 0.0001) at day +1 (Figure 6H). ROC curves showed that a value above 181.5 of CAR^+^EVs/μL at hour +1 predicted the onset of grade ≥2 ICANS with a sensitivity of 75.00% and specificity of 82.09% (*P* < 0.0001), while a value above 372.3 CAR^+^EVs/μL at day +1 was able to predict the onset of grade ≥2 ICANS with a sensitivity of 85.00% and specificity of 81.54% (*P* < 0.0001) (Figure 6I). Notably, CAR^+^EVs/μL levels were positively correlated with the expansion peak levels of CD8^+^CAR^+^T effector memory cells (CD8^+^CAR^+^T_EM_; hour +1 CAR^+^EVs: *r* = 0.580, *P* = 0.002; day +1 CAR^+^EVs: *r* = 0.463, *P* = 0.017) (Supplemental Figure 6, A and B). Similar numbers of total plasma EVs were found in the plasma of patients developing ICANS and NO ICANS at hour +1 and day +1, suggesting that the above-reported phenomenon is specific to the CAR^+^EV compartment (Supplemental Figure 6, C and D). We then tested whether early CAR^+^EV levels were correlated also with the onset of any grade of CRS. Even though no differences were found in hour +1 CAR^+^EV median levels (CRS vs. NO CRS, 113 vs. 125 CAR^+^EVs/μL, Mann-Whitney test, *P* = 0.596), higher levels of day +1 CAR^+^EV median levels were found in CRS compared with NO CRS patients: 223 (range 65–4,012) versus 151 (range 56–251) CAR^+^EVs/μL (Mann-Whitney test, *P* = 0.026) (Supplemental Figure 7, A–D). However, according to logistic regression analysis, neither hour +1 nor day +1 CAR^+^EVs could be taken as CRS predictors (OR = 1.004, 95% CI: 0.99–1.01, *P* = 0.26, for hour +1 CAR^+^EVs/μL; OR = 1.01, 95% CI: 1.00–1.02, *P* = 0.17, for day +1 CAR^+^EVs/μL). Nevertheless, in patients with grade ≥2 CRS, we observed higher median levels of CAR^+^EVs compared with grade 0–1 CRS: 271.5 (range 29–1,944) versus 108 (range 37–831) CAR^+^EVs/μL (Mann-Whitney test, *P* = 0.034) at hour +1; 682 (range 68–4,012) versus 177.5 (range 56–2,042) CAR^+^EVs/μL (Mann-Whitney test, *P* = 0.007) at day +1 (Supplemental Figure 7, A–D). Hence, high plasma CAR^+^EV levels can be taken as reliable early markers of ICANS onset and severity, while the role of CAR^+^EVs in predicting CRS is less evident.

### Neuron-specific ENO2-positive nanoparticles as markers of CAR^+^EV-induced neural stress.

**Figure 7:**
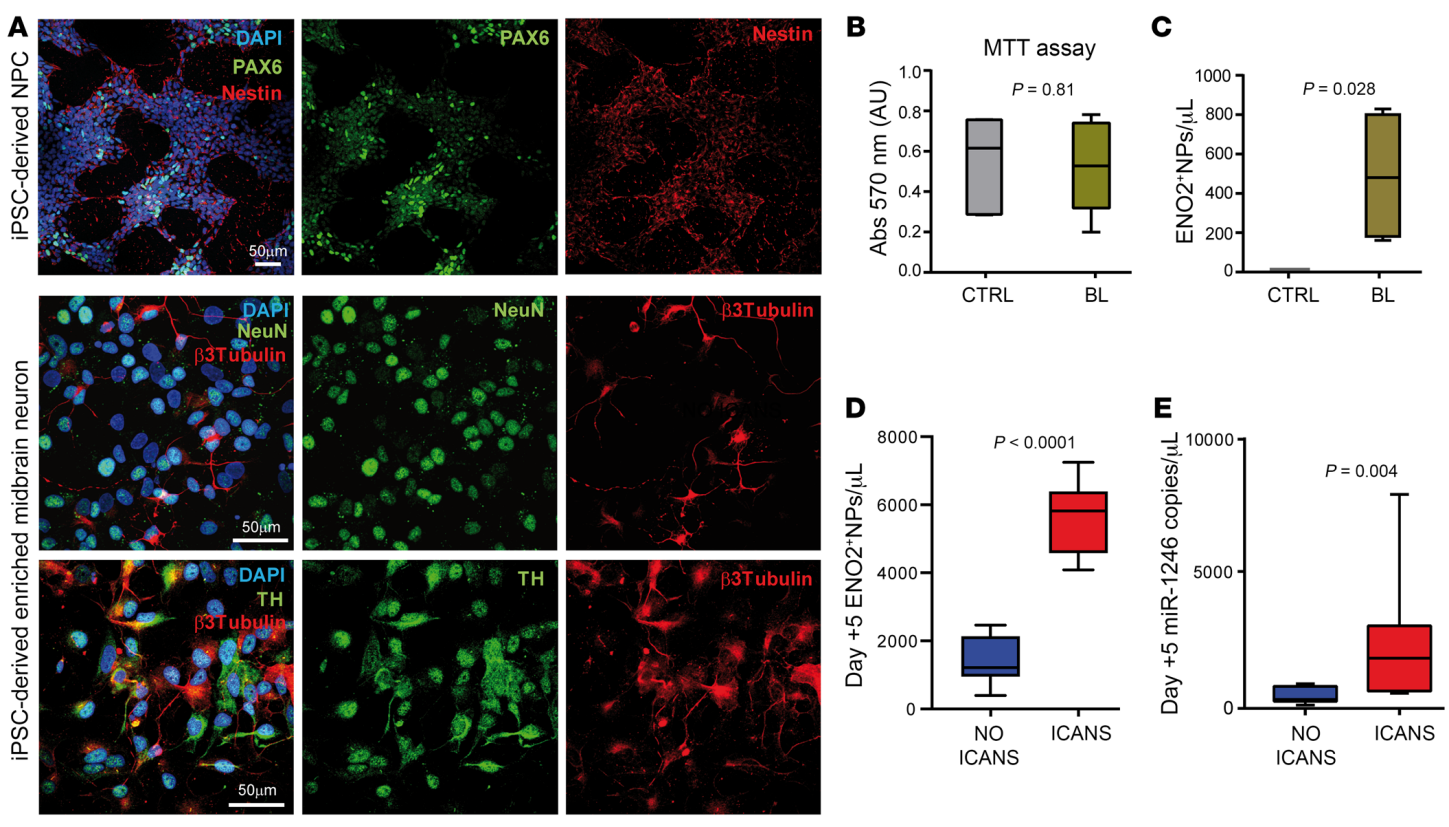
Neuron-specific Enolase 2–positive NPs: markers of neural damage. (**A**) Representative pictures of healthy donor iPSC-derived neural cells: neural precursor cells (NPCs) stained with Abs against Pax6 (green) and nestin (red), and enriched midbrain neurons stained with Abs against β_3_-tubulin (red) and NeuN (green) or Abs against tyrosine hydroxylase (TH; green) and β_3_-tubulin (red). Nuclei were counterstained with DAPI (blue) (*n* = 3). Scale bars: 50 μm. (**B**) MTT assay of iPSC-derived neural cells exposed to EVs from CD19^+^ cell supernatants (CTRL, *n* = 6) versus BL (*n* = 14). (**C**) MFC analysis of Enolase 2–positive (ENO2^+^) NP release by iPSC-derived neural cells exposed to EVs from CD19^+^ cell supernatants (CTRL, *n* = 4) versus BL (*n* = 4); MW test. (**D** and **E**) Day +5 analysis of plasma ENO2^+^NPs assessed by MFC (**D**) and miR-1246 assessed by ddPCR (**E**) in ICANS (*n* = 8) versus NO ICANS (*n* = 12); MW test. Data are presented as boxes and whiskers; boxes show median and IQR, and whiskers represent minimum and maximum values.

Owing to the association of CAR^+^EVs with ICANS, we tested the hypothesis that CAR^+^EVs may mediate toxic effects on neural cells. To this aim, we exploited an in vitro model of human iPSC-derived neural progenitors and mature neurons (Figure 7A). CAR^+^EVs purified from 19BBζ.CAR T cells and two CD19.CAR T BLs exerted negligible effects on iPSC-derived neuron viability and/or metabolic activity (Figure 7B). As extracellular ENO2 has been reported to be a marker of neural stress (38, 39) and because Zhang et al. (25) recently reported ENO2 to be part of subcellular structures, called supermeres, characterized as extracellular nanoparticles (NPs), we sought to check ENO2^+^NP release in the supernatant of iPSC-derived neurons exposed to CAR^+^EVs. Upon 24-hour exposure to CAR^+^EVs, we found measurable amounts of ENO2^+^NPs in the iPSC supernatants, but not in the ones with untreated cultures (Figure 7C). To confirm the role of ENO2^+^NPs as markers of ongoing CAR T–related neurotoxicity, we assessed the plasma of 8 ICANS and 12 NO ICANS patients at day +5, which represents the median day of ICANS onset. We found higher plasma levels of ENO2^+^NPs in patients with ICANS compared with NO ICANS ones (*P* < 0.0001; Figure 7D). Notably, Zhang et al. also reported that ENO2^+^NPs carry abundant amounts of miR-1246 (25). Indeed, we demonstrated that median plasma levels of miR-1246 were higher in ICANS than in NO ICANS patients (1,797 vs. 240.4, *P* = 0.0041; Figure 7E), and that ENO2^+^NPs and miR-1246 plasma levels were positively correlated (*r* = 0.521, *P* = 0.018), suggesting that ENO2^+^NPs and miR-1246 could be markers of neural stress in CD19.CAR T cell–infused patients developing ICANS.

## Discussion

This study was inspired by the compelling need to identify early CAR T cell therapy–induced neurotoxicity biomarkers, which could also provide new insights into ICANS pathogenesis. We identified the release of CAR^+^EVs as an immediate signal of CD19.CAR T cell activation in vitro, as CAR^+^EVs were mostly released upon the engagement of the cognate target cells. CAR^+^EVs were also detectable in the plasma of CAR T cell–infused patients as soon as 1 hour after infusion. In vitro release and circulating plasma CAR^+^EVs were characterized by ExoView platform, MFC, and STORM imaging. These techniques were able to demonstrate the presence of exosome markers (predominantly CD63 protein) (40) on CAR^+^EVs. Moreover, combined and correlative confocal/STORM analysis showed that most CAR^+^EVs are likely to be preassembled as CD63-rich granules in the cytoplasm of CD19.CAR T cells (35). MFC analysis also revealed that CAR^+^EVs are devoid of CD45 protein, but enriched in TCR-associated molecules (41), such as CD8, CD4, and the CD3ζ-associated ZAP70, thus underpinning their possible relationship with the kinetics of nano-particles and nano-vesicles released during the engagement of the immunological synapse (35, 42). Notably, MFC analysis detected a rapid appearance of a double-positive EV population (CAR^+^CD19^+^EVs) after CAR T cell/target interaction (Supplemental Figure 8A), recalling the phenomenon of trogocytosis, i.e., the nibbling of the cell membrane after ligand/receptor interaction (43, 44). In this regard, we found CD19 protein on CAR^+^EVs released after the CD19^+^ target engagement, but not on CAR^+^EVs released before target engagement, or by CD19.CAR T cells challenged with CD19^−^ cells (Supplemental Figure 8B). Consistently, CD19 protein was present on CAR^+^EVs in the plasma of CAR T cell–infused patients at different time points (Supplemental Figure 8C). Although the significance of CAR^+^CD19^+^EVs warrants further investigation, these data support the notion that CAR^+^EVs should be taken as potential markers of CD19.CAR T cell target engagement and activation. As further confirmation that CAR^+^EV plasma levels are a reliable readout of in vivo CAR T cell activation, day +1 CAR^+^EVs were positively correlated with day +7 circulating CD19.CAR T cells and CAR^+^CD8^+^T_EM_ peak levels. Furthermore, in the early hours after infusion, plasma CAR^+^EV levels become detectable, paralleling the disappearance of circulating CAR T cells (measured by the drop of CAR_DNA copies in the whole blood). This phenomenon was more prominent in patients who later developed ICANS, suggesting a higher activation rate of CD19.CAR T cells. Patients with ICANS also showed elevated plasma levels of IL-15, an extensively characterized CAR T cell activator (45–47), and CX3CL1, an acknowledged mediator of T cell homing (48). Moreover, IL-15 and CX3CL1 plasma levels were positively correlated with markers of inflammation and high tumor burden (fibrinogen, lactate dehydrogenase, C-reactive protein, cell-free DNA, CXCL9) at pre-lymphodepletion, a biochemical makeup that has been associated with ICANS (14, 20). Notably, higher levels of day +1 CX3CL1 and IL-15 were found in patients receiving chemotherapy drug–based bridging therapy. Hence, our data support the notion that ICANS is more likely to occur in the presence of enhanced CD19.CAR T cell activation in the first hours after infusion and may be related to the activity of IL-15 and CX3CL1 pathways. Following this reasoning, plasma CAR^+^EVs turned out to be a reliable predictor of ICANS, as they were easily measurable in the plasma 4 days before the median (day +5) onset of neurotoxicity. Instead, the in vivo expansion of CD19.CAR T cells was an unsuitable predictor of ICANS, as it reached its peak later than the median onset of the first neurological symptom. In accordance with current literature (11, 12, 15), we found that high-grade CRS is tightly associated with ICANS also in our case set (Table 1). However, in spite of higher day +1 CAR^+^EV levels in patients developing CRS, CAR^+^EVs turned out to be unsuitable predictors of its occurrence, owing to the overlapping with the median (day +1) onset of CRS. Data from the literature suggest that CAR^+^EVs may kill cells that carry their target molecule (23, 24). In regard to this issue, CAR^+^EVs were found to carry high amounts of PRF1, thus being potentially toxic for the CNS (49). In particular, the lack of endosomal marker CD107a and the presence of the wheat germ agglutinin–detectable glycoprotein content recall the supramolecular complexes that contain PRF1 and are expected to be endowed with autonomous extracellular killing activity (36). Intriguingly, CAR^+^EVs exert negligible toxicity on iPSC-derived neural cells, but elicit the release of ENO2, a marker of neural stress (38, 39). This latter was measurable by MFC as an EV-like structure that recalls a class of NPs named supermeres. These NPs are enriched in miR-1246, can be detected in the plasma, and can potentially pass through the blood-brain barrier (25). Strikingly, high levels of ENO2^+^NPs and of miR-1246 were found in the plasma of patients with ICANS, suggesting their potential role as markers of neural cell stress/toxicity. Overall, this paper shows that CAR^+^EVs are markers of CAR T cell activation and are early predictors of ICANS. Their role in the pathogenesis of neurotoxicity warrants further investigation.

## Methods

Full description of methods is reported in Supplemental Methods and Supplemental Figure 9, A–D.

### Sex as a biological variable.

All consecutive patients were enrolled regardless of gender; both sexes were included in the study, and the variable “sex” was included in the statistical analysis.

### Study design.

This is a prospective observational tissue study aimed at discovering potential biomarkers predictive of either response or complications in patients affected by relapsed/refractory B cell lymphoma after CD19.CAR T cell therapy, using Italian Medicines Agency (AIFA)–approved advanced cell therapy products (tisagenlecleucel, axicabtagene ciloleucel, and brexucabtagene autoleucel). All eligible patients were treated at IRCCS Azienda Ospedaliero–Università di Bologna (IRCCS AOU di Bologna), after signing a written informed consent. The studies were approved by the Ethics Committee, registered at ClinicalTrials.gov (NCT04892433, NCT05807789), and run in agreement with the Declaration of Helsinki.

### Sampling and assessment of biochemical parameters.

Blood samples obtained by venipuncture were collected in 10 mL EDTA tubes (BD Vacutainers, 36752). Sampling was performed according to the following schedule: pre-lymphodepletion (ward admission) and 1 hour and 1, 3, 5, 7, 9, 11, 13, 21, and 30 days after CD19.CAR T cell infusion. Plasma separation was performed by centrifugation at 1,500*g* for 15 minutes at room temperature. Plasma and blood aliquots were immediately collected and stored at –80°C. All the serum biochemistry reported in the study was assessed according to the standard practice at the IRCCS AOU di Bologna analysis service.

### Graphical abstract.

The graphical abstract was realized with BioRender (individual license GC26IQM3IO to GS).

### MFC analysis and sorting of CAR^+^EVs.

Plasma, bag leftovers (BLs), and cell culture supernatant–derived EVs were assessed and quantified by cytofluorimetric assays performed on different platforms, i.e., FACSLyric (BD Biosciences) and CytoFLEX (Beckman Coulter). On the former platform, “protocol 1” refers to that previously described (50, 51). Briefly, 5 μL of patients’ serum was added to 95 μL of 0.1-μm-filtered PBS and 95 μL of a mix of anti-human mAbs: CD31–PE-Cy5.5, clone WM-59 (BD Biosciences catalog 563651, RRID:AB_2738348); CD45-BV510, clone HI30 (BD Biosciences catalog 563204, RRID:AB_2738067); and CD41a-PE, clone HIP8 (BD Biosciences catalog 557297, RRID:AB_396624). FITC-conjugated phalloidin and APC-conjugated lipophilic cationic dye (Dye Integer EV Detection Kit, BD Biosciences catalog 626267) were used to exclude non-EV events from the analysis. The number of EVs per microliter was assessed using the TruCount BD system (BD Biosciences). Protocol 2 (52) was set up using Megamix-Plus FSC microparticles of known size (BioCytex, STAGO group, catalog 7802). Briefly, gating strategy was set by Megamix, and the particle size of each region was assessed following the manufacturer’s instructions. The EV phenotype was subsequently analyzed by differentiation of the EVs on the basis of the size-corresponding region. The Abs used in both methods were PE-conjugated-CD19 CAR FMC63 Idiotype Ab (Miltenyi Biotec catalog 130-127-342) or APC-conjugated-CD19 CAR FMC63 Idiotype Ab, clone REA1297 (Miltenyi Biotec catalog 130-127-343) and the following fluorochrome-conjugated anti-human mAbs: CD63-BV510, clone H5C6 (BD Biosciences catalog 740182, RRID:AB_2739935); perforin–PerCP-Cy5.5, clone δG9 (BD Biosciences catalog 563762, RRID:AB_2738409); FAS ligand–PE, clone NOCK-1 (BD Biosciences catalog 564261, RRID:AB_2738713); CD107a–APC-H7, clone H4A3 (BD Biosciences catalog 561343, RRID:AB_10644020); CD8β-PE, clone 2ST8.5H7 (BD Biosciences catalog 641057, RRID:AB_1645747); CD19–PE-Cy7 clone SJ25C1 (BD Biosciences catalog 557835, RRID:AB_396893); ZAP70-FITC, clone 1E7.2 (BD Biosciences catalog 344934, RRID:AB_647380); CD151–Alexa Fluor 488, clone 210127 (R&D Systems); ENO2-PE, clone ENO2/1375 (Novus Biologicals, Bio-Techne). Protocol 2 was performed whenever the utilized mAbs were conjugated to fluorochrome, interfering with protocol 1. A representative sample assessed with the 2 protocols is reported in Supplemental Figure 10, A and B. CytoFLEX SRT equipment (Beckman Coulter) was used to sort CAR^+^EVs. Briefly, protocol 2 and side scatter (SSC) violet laser were combined for EV gating strategy. Sorting regions were defined by fluorescent markers, and 10^6^ events were collected in the final tube and stored at –80°C (Supplemental Figure 10C).

### Confocal microscopy analysis.

CAR T cells or iPSC-derived neural cells were spun onto coverslips or seeded on cover-glasses (Corning) coated with Matrigel (Corning), respectively, fixed with 4% paraformaldehyde for 15 minutes at room temperature, rinsed twice with PBS, blocked, and permeabilized with 1% BSA in 0.1% Triton X-100 for 1 hour at room temperature. After saturation of nonspecific binding with 4% BSA/PBS solution for 20 minutes, coverslips were incubated with primary-labeled or click-conjugated Abs at room temperature for 1 hour. The following products were used for CAR T cell stainings: Zenon Alexa Fluor 555 mouse IgG1 labeling kit (Thermo Fisher Scientific catalog Z25005, RRID:AB_2736948) and Zenon Alexa Fluor 647 human IgG labeling kit (Thermo Fisher Scientific catalog Z25408, RRID:AB_2736958). The mAbs used were perforin, clone eBioBOR21 (Thermo Fisher Scientific catalog 14-9993-82, RRID:AB_468675); CD63, clone TS63 (Thermo Fisher Scientific catalog 10628D, RRID:AB_2532983); CD19 CAR FMC63 Idiotype Ab, clone REA1297 (Miltenyi Biotec catalog 130_127_983); and DAPI [2-(4-amidinophenyl)-6-indolecarbamidine, Thermo Fisher Scientific catalog 62247] for nucleic acid staining. The following Abs were used for iPSC-derived neural precursor cells and neurons: Pax6 (Thermo Fisher Scientific catalog 42-6600, RRID:AB_2533534), nestin (Thermo Fisher Scientific catalog MA1-110, RRID:AB_2536821), NeuN (Abcam catalog ab190565, RRID:AB_2732785), β_3_-tubulin (Abcam catalog ab78078, RRID:AB_2256751), tyrosine hydroxylase (GeneTex catalog GTX113016, RRID:AB_1952230). After 3 PBS washes, samples were stained with the appropriate secondary fluorochrome-conjugated Abs: Alexa Fluor 488–conjugated goat anti-rabbit (Abcam catalog ab150077, RRID:AB_2630356), Alexa Fluor 555–conjugated goat anti-mouse (Abcam catalog ab150114, RRID:AB_2687594), Alexa Fluor 647–conjugated donkey anti-mouse (Abcam catalog ab150107, RRID:AB_2890037), for 2 hours at room temperature. Samples were mounted with anti-fade reagent (Molecular Probes Life Technologies, Milan, Italy). Confocal images were acquired with a Nikon A1R laser scanning confocal microscope equipped with an Eclipse Ti-E inverted microscope and 4 laser lines (405, 488, 561, and 638 nm) (Nikon). *Z*-series images were taken with an inter-stack interval of 0.3 μm using a ×100 total internal reflection fluorescence (TIRF) (NA 1.49) objective. Laser intensity and detector gain were maintained constant for all images within the same set of experiments. Confocal images were processed using Richardson-Lucy deconvolution algorithm. Image processing, 3D rendering, and colocalization analysis were obtained using the software NIS-Elements v5.31 (Nikon) as previously described (53). For time-lapse confocal imaging, fluorescence image series were acquired with a Nikon A1R confocal microscope with a ×20 NA 0.75 Plan Apo VC objective. All experiments were carried out at 37°C and 5% CO_2_ using a stage incubation (OkoLab) and an Eclipse Ti-E inverted microscope (Nikon) equipped with a perfect focus system. 489.1 nm and 561 nm diode lasers were used for excitation of PKH26 Red Fluorescent Cell Linker Kit (Sigma-Aldrich catalog PKH26GL) and Sytox Green nucleic acid stain (Thermo Fisher Scientific catalog S7020) or eGFP, respectively, and set on less than 10% to minimize the possible phototoxic effects induced by fluorescence illumination on live cells. The diameter of the detection pinhole was set at 2 Airy units (59 μm) to generate a single thick optical section passing through the center of the cells. A series of sequential images of 1,024 × 1,024 pixels at 12 bits (4,096 gray levels) were collected at a fixed pixel size of 63 μm, every 5 minutes for at least 16 hours. The transmission and fluorescence images were merged and rendered using NIS-Elements Advanced Research software (Nikon).

### STORM analysis and combined confocal microscopy/STORM analysis.

Single-molecule super-resolution microscopy for CD19.CAR antigen was performed as previously described (54). Acquisitions for single-channel detection were performed on an N-STORM instrument (Nikon) equipped with a DU-897 EM-CCD camera (Andor Technology), with ×100 TIRF (NA 1.49) objective and coupled with a 10-mW 647-nm-excitation/reported laser (CrystaLaser) used at 80% power for 10,000 frames per acquisition over a constant TIRF-plane angle, with 1-frame-exposure detection (at 10- to 20-millisecond range), for both 2D- and 3D-STORM. Data reconstruction was obtained with the STORM analysis module of the NIS-Elements software v5.31 (Nikon). Samples were stained with Alexa Fluor 647–conjugated human recombinant CD19.CAR FMC63 Ab Idiotype (Miltenyi Biotec), incubated overnight at 4°C, and washed with PBS. Before image acquisition, blinking direct STORM (dSTORM) buffer was added, and samples were covered with 1.5H coverslips. Where indicated, after confocal detection, the anti-fading mounting solution was replaced with STORM buffer to obtain a proper blinking for dSTORM analysis, as illustrated in the experimental scheme. Dual-color STORM acquisition was performed on an Olympus Ix83 inverted microscope (Evident) with a ×100 NA 1.5 TIRF objective to which an Abbelight SAFe MN360 platform was attached equipped with 405-nm-, 488-nm-, 561-nm-, and 640-nm-wavelength lasers (Oxxius). 19BBζ.eGFP.CAR T cells were spun on glass coverslips and fixed as previously described, stained with Alexa Fluor 568–conjugated mAb CD63 (contained in EV Profiler kit, ONI) and Alexa Fluor 647–conjugated human recombinant CD19.CAR FMC63 Ab Idiotype (Miltenyi Biotec), and acquired in simultaneous multicolor dSTORM (laser power 640 nm, 300 mW, and 561 nm, 165 mW, for 10,000 frames per acquisition over a constant HiLo-plane angle and 50 milliseconds of exposure time). eGFP channel was acquired as a wide-field image (laser power 488 nm, 40 mW, 500 milliseconds of exposure time). Both eGFP^+^ and eGFP^–^ EVs were captured on tetraspanin-coated glasses according to Smart EV kit protocol (Abbelight). eGFP EVs were labeled with a pool of CD63, CD9 and CD81 Abs (Abbelight) all conjugated with Alexa Fluor 647 and imaged as monocolor 2D-STORM (laser power 640 nm, 300 mW, and 405 nm, uniform gradient from 0 to 40 mW during the entire acquisition, for 10,000 frames per acquisition over a constant HiLo-plane angle and 50 milliseconds of exposure time). Plasma CAR^+^EVs were labeled with Alexa Fluor 647–conjugated human recombinant CD19.CAR FMC63 Ab Idiotype (Miltenyi Biotec) and CF680-conjugated rabbit recombinant CD63 EPR21151 Ab Idiotype (Abcam) and imaged in Spectral demixing dSTORM (laser power 640 nm, 300 mW, and 405 nm, uniform gradient from 0 to 40 mW during the entire acquisition, dichroic mirror T700 for 10,000 frames per acquisition over a constant HiLo-plane angle and 50 milliseconds of exposure time). The acquired single-molecule blinking videos were super-localized with NEO_analysis software (Abbelight) to obtain super-resolution coordinate tables. Channel-alignment spectral demixing was performed in NEO_analysis (Abbelight). Single-EV segmentation was performed using DBSCAN in NEO_analysis (Abbelight) and is described in Supplemental Methods. EV sizing and color assignment were performed with custom Python scripts, and data are available in Supplemental Methods and in the Supporting Data Values file.

### ExoView platform analysis.

Whole CAR T patient plasma EVs, infusion BL EVs, and EVs isolated by SmartSEC columns were analyzed by ExoView platform (NanoView Biosciences) following the manufacturer’s protocol. Abs used for EVs detection were: recombinant human PE-conjugated CD19 CAR FMC63 Ab Idiotype (Miltenyi Biotec); fluorochrome-conjugated anti-human mAbs CD107a–APC-H7, clone H4A3 (BD Biosciences catalog 561343, RRID:AB_10644020), and perforin–Alexa Fluor 647, clone dg9 (BioLegend catalog 308110, RRID:AB_493254); and CD9–Alexa Fluor 488, CD81–Alexa Fluor 555, and CD63–Alexa Fluor 647 provided by NanoView Biosciences. Recombinant human CD19 Fc Chimera Alexa Fluor 647 Protein (Bio-techne, catalog AFR9269) was employed. Alexa Fluor 488 wheat germ agglutinin was purchased from Invitrogen (Thermo Fisher Scientific). The analysis was performed according to the manufacturer’s instructions using the ExoView R200 reader endowed with ExoView Scanner software (v3.0).

### Statistics.

Descriptive statistics are reported for the whole population as required. All tests were performed as 2-tailed, and *P* values less than 0.05 were considered significant. The association of clinical and laboratory variables with ICANS was assessed through Pearson’s χ^2^ test, Mann-Whitney *U* test, and 1-way ANOVA, depending on categorical, non-normal, and normal distribution, respectively. All variables significantly (*P* ≤ 0.05) associated with ICANS were evaluated in multiple logistic regression models. Student’s *t* test was performed for log-transformed fold changes (logFC) at each time point; *P* values were considered significant according to Benjamini-Hochberg correction for multiple comparisons. Alternatively, paired *t* test and 1-sample *t* test were applied where needed. Median time to CAR^+^T cell expansion peak (TTP) was calculated according to the Kaplan-Meier estimator. Correlation among variables was measured by means of either Spearman’s or Pearson’s coefficient. Confocal and STORM microscopy colocalization analysis was performed by calculation of Manders’ coefficient as previously described (55). All statistical analyses were performed with SPSS (RRID:SCR_002865, IBM Corp.), GraphPad Prism (RRID:SCR_002798 Dotmatics), and R, freely available from the Comprehensive R Archive Network (r-project.org).

### Study approval.

The study was approved by the institution: Comitato Etico di Area Vasta Emilia Centro della Regione Emilia-Romagna (approval 319/2021/Sper/AOUBo and EM714-2022_319/2021/Sper/AOUBo).

### Data availability.

Python code script is reported in Supplemental Methods. All underlying data are reported in the Supporting Data Values file.

## Author contributions

GS and FDF share first authorship: GS hypothesized the presence and the role of CAR^+^EVs and presided over laboratory data and in vitro experiments; FDF presided over the management of clinical data and statistical analysis. In particular, GS conceptualized and designed all the experiments and analysis on CAR^+^EVs and ENO2^+^NPs, was in charge of performing experiments, followed the analytical process of all the samples, and prepared the figures as well as cowrote the manuscript and therefore was listed first. FDF designed and compiled the clinical database of the patients involved in this study, prepared the figures, performed the statistical analysis, and cowrote the manuscript. MB and F Bonifazi share last authorship: MB is the principal investigator of the preclinical research group, and F Bonifazi is the principal investigator of the clinical group and of the Laboratory of Immunobiology of Transplant and Cellular Therapies. MB and F Bonifazi are the holders of the research grants that allowed this study to be carried out. GS, FDF, SDM, SNB, MB, and F Bonifazi conceived and designed the study. GS, SNB, SS, FR, PLT, NL, MF, MN, SDM, DM, LR, FV, and CS developed methodologies. GS, FDF, FR, DM, MD, MU, F Barbato, MR, MG, GMA, CC, MA, PC, EM, ET, NL, MN, BC, C Pellegrini, C Pirazzini, SG, MC, ED, BS, CP, PG, KMK, PLZ, F Bonifazi, MT, and CS acquired data. GS, FDF, MB, F Bonifazi, SS, FI, and CS analyzed and interpreted results. GS, FDF, DM, MB, and F Bonifazi wrote and revised the manuscript. GS, FDF, MD, ET, MN, and NL provided administrative, technical, or material support. MB, PLZ, and F Bonifazi supervised the study. MG, GMA, and PC performed neuropathological assessment.

## Supplementary Material

Supplemental data

ICMJE disclosure forms

Supplemental video 1

Supplemental video 2

Supporting data values

## Figures and Tables

**Table 1:**
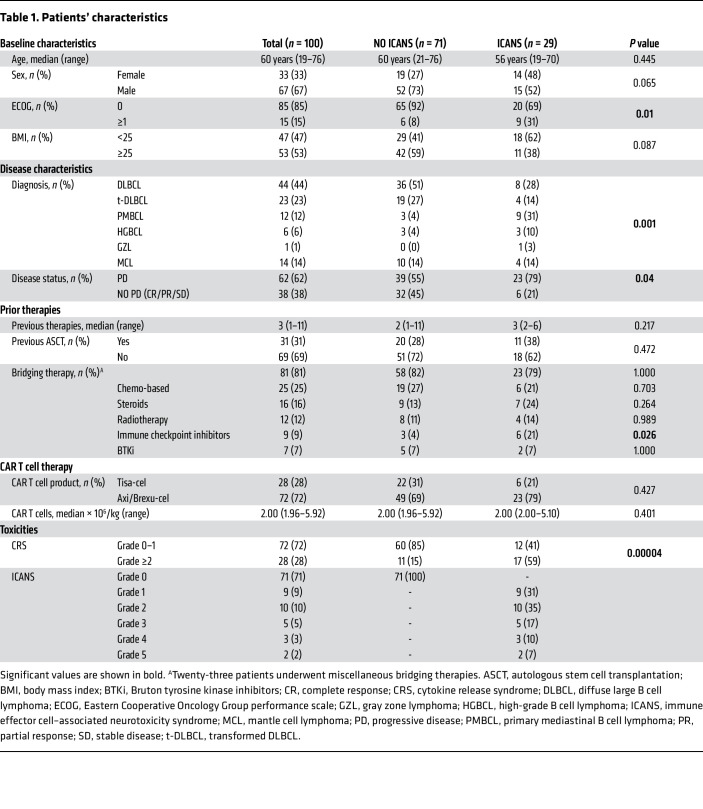
Patients’ characteristics

## Version 1 06/04/2024

In-Press Preview

## Version 2 07/15/2024

Electronic publication
