# Supplementary material for: CAR+ extracellular vesicles predict ICANS in patients with B cell lymphomas treated with CD19-directed CAR T cells
Source: J Clin Invest. 2024 Jun 4;134(14):e173096. doi: 10.1172/JCI173096 (PMC11245152; doi:10.1172/JCI173096)
Supplement: Supplemental data [file jci-134-173096-s115.pdf]

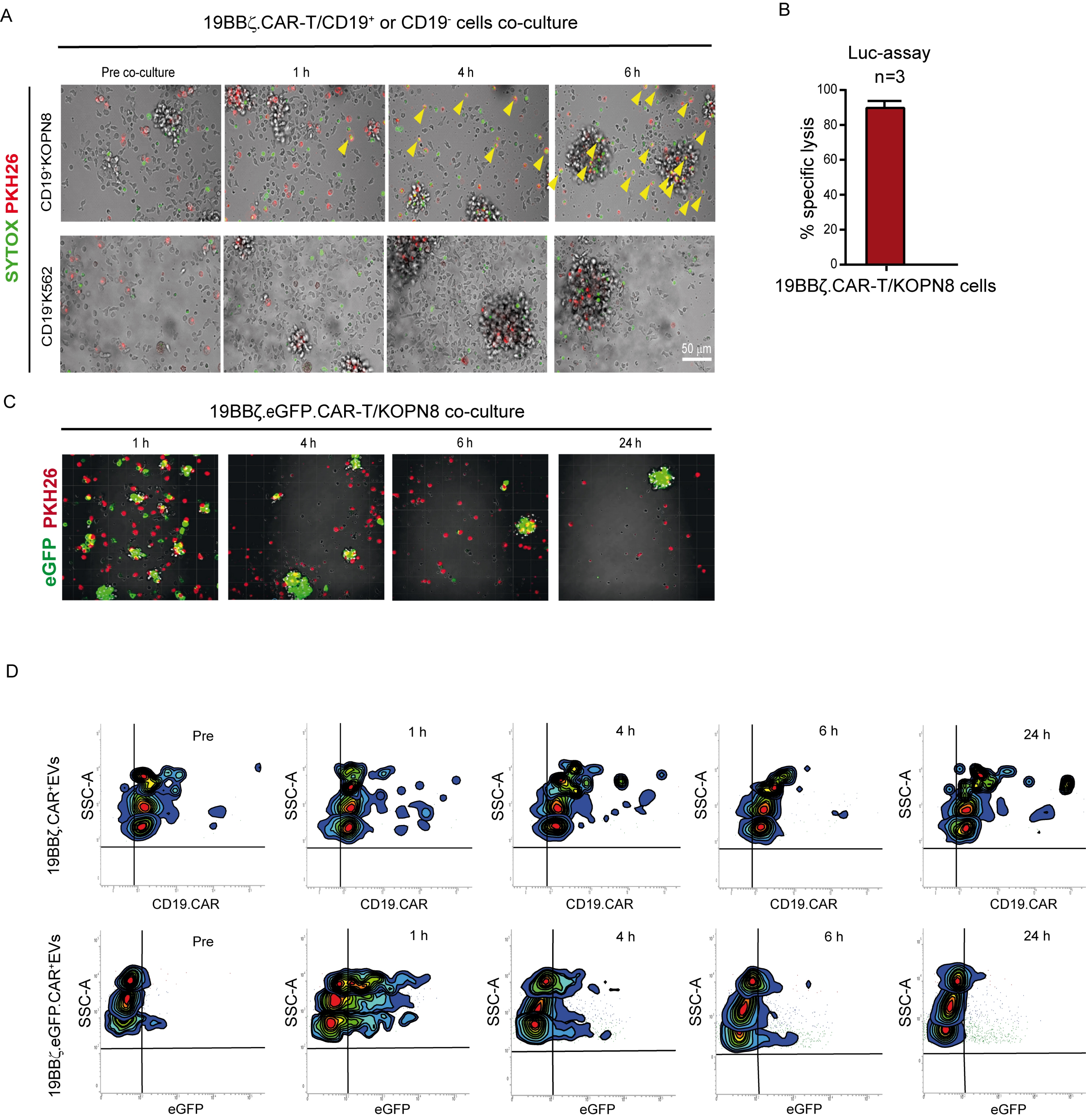

Revised_Supplementary Figure 1

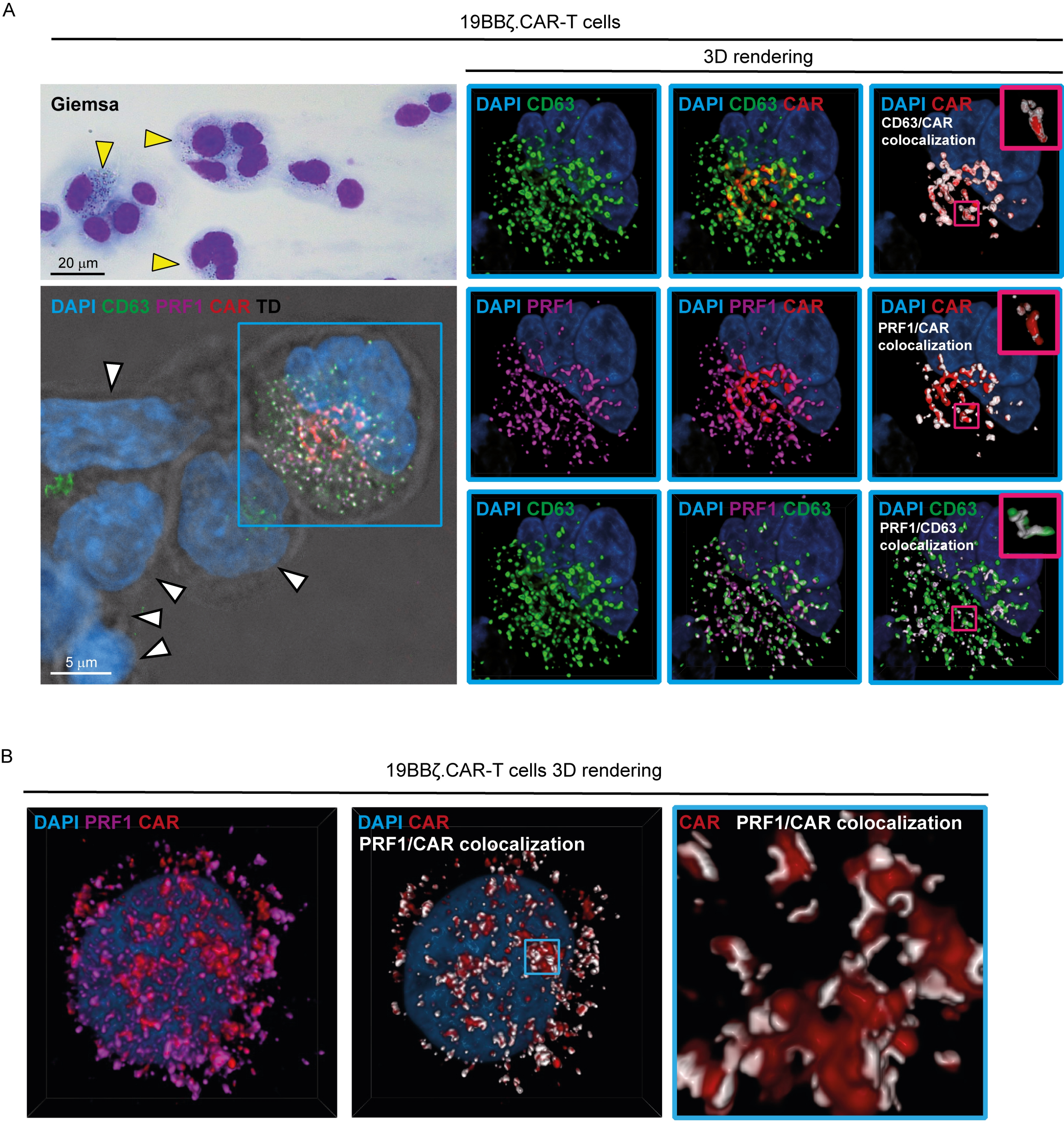

Revised Supplementary Figure 2

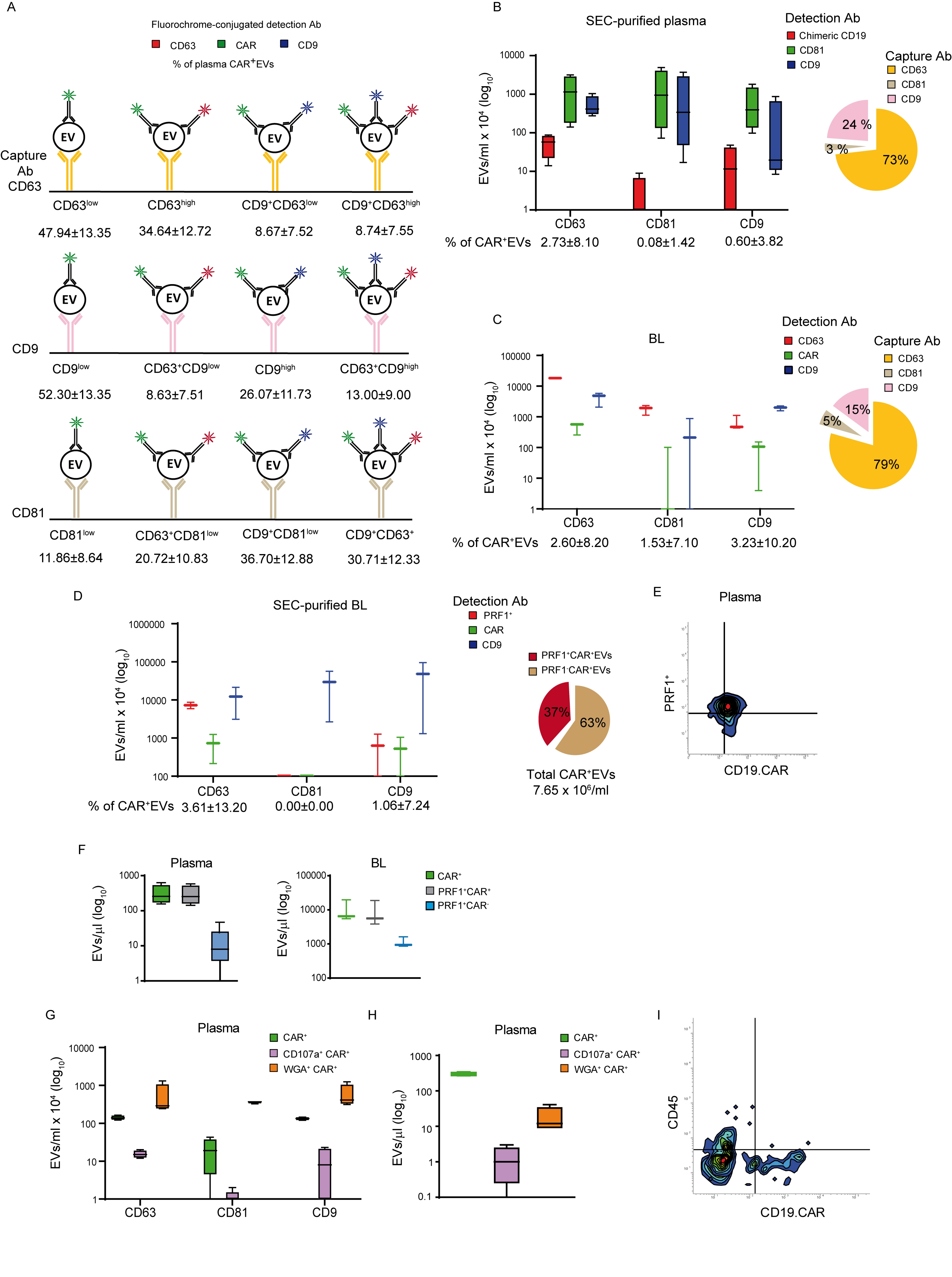

Revised supplementary Figure 3

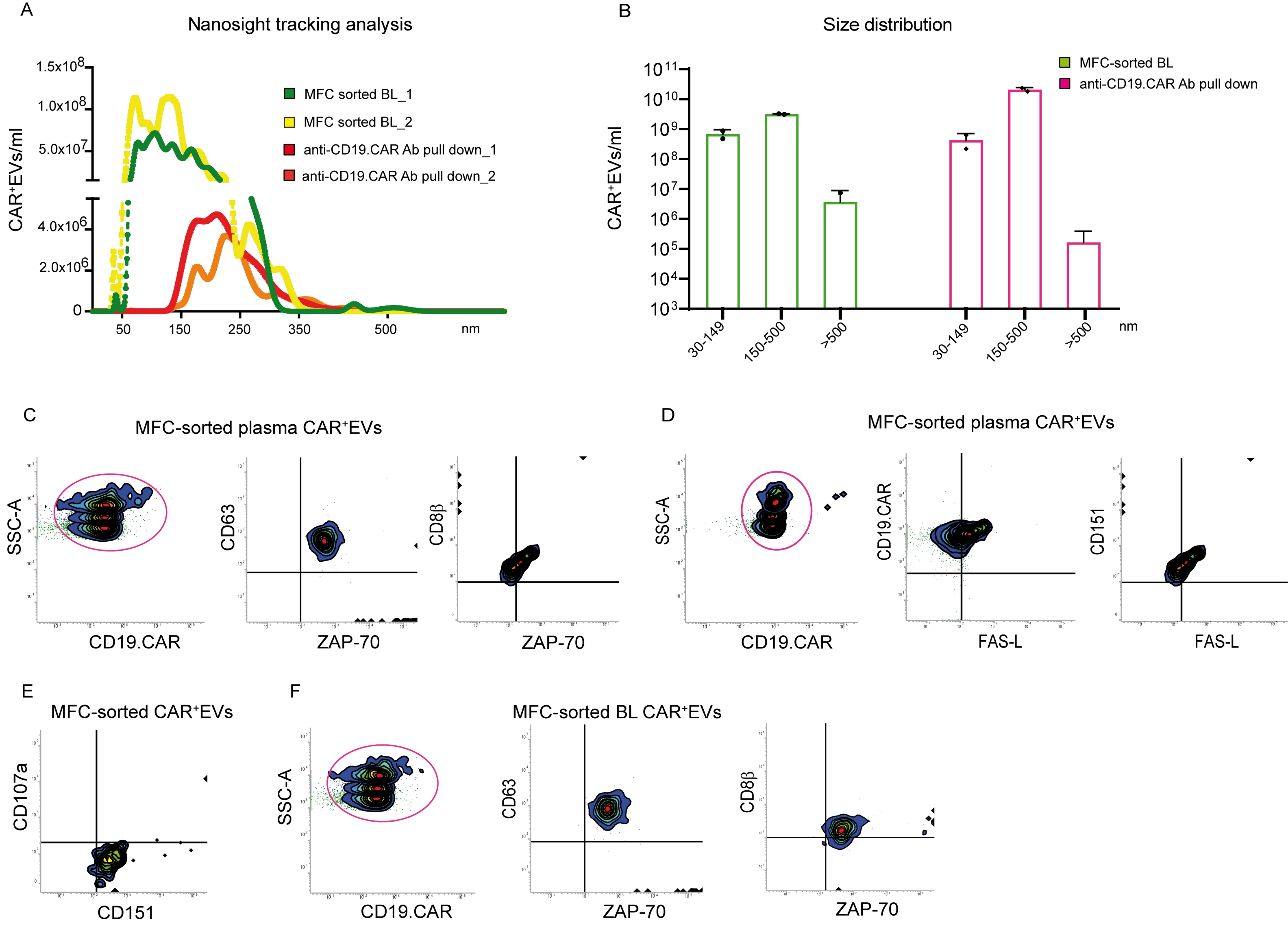

Revised Supplementary Figure 4

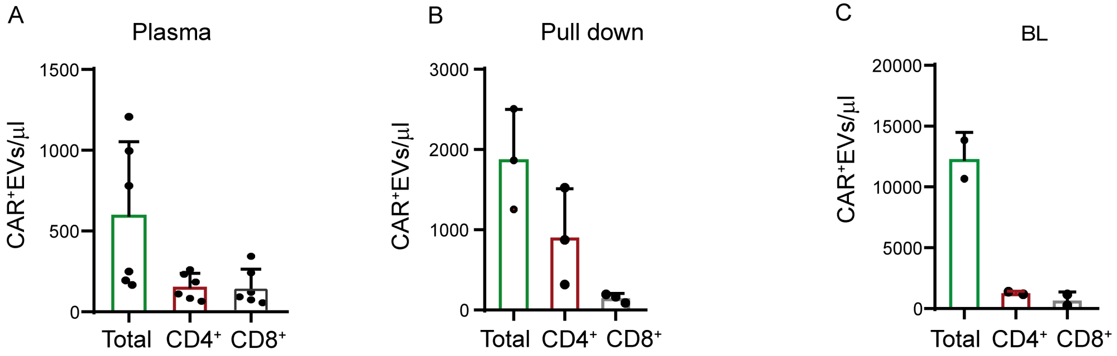

Revised Supplementary Figure 5

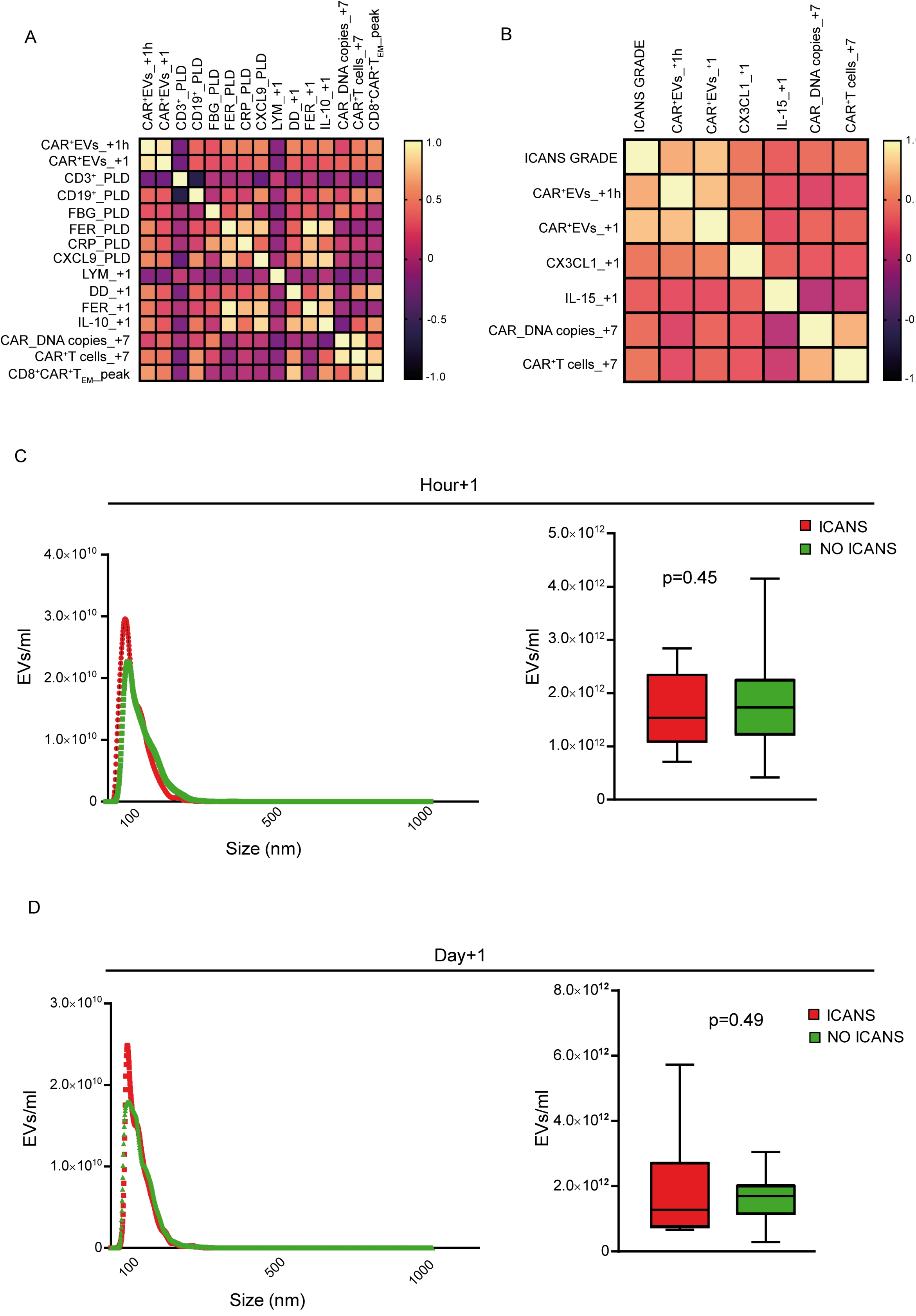

Revised Supplementary Figure 6

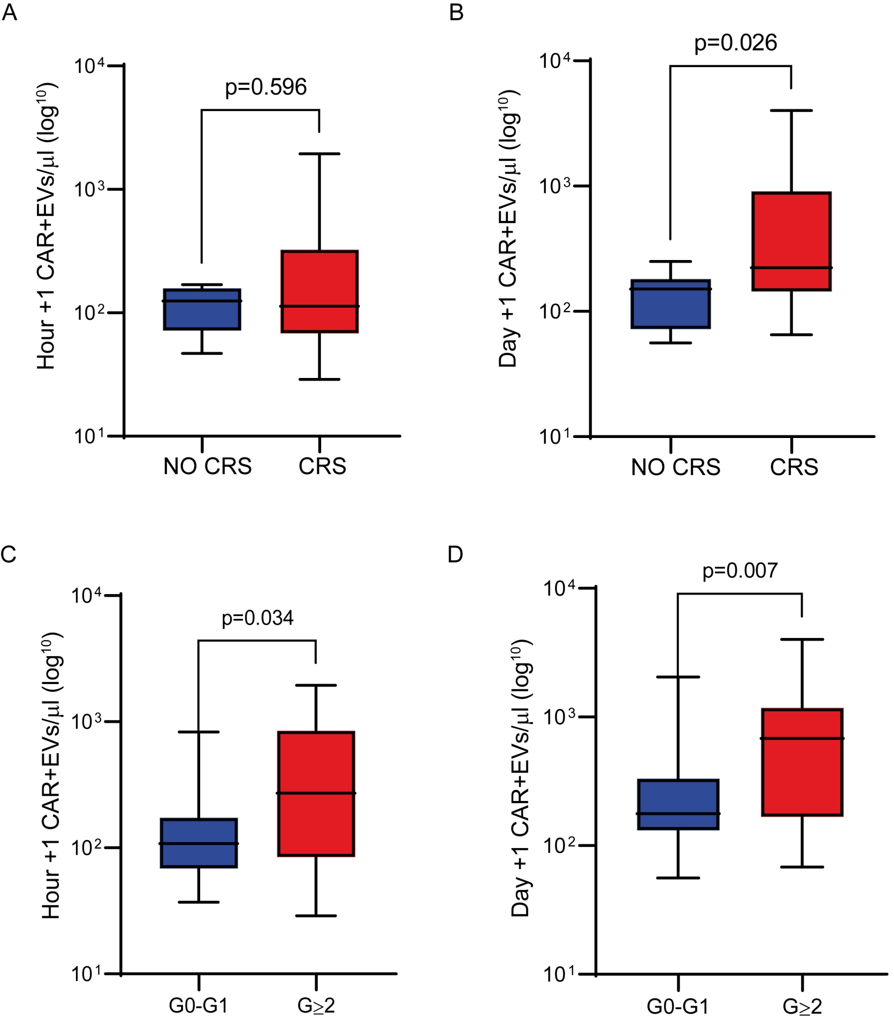

Revised_Supplementary figure 7

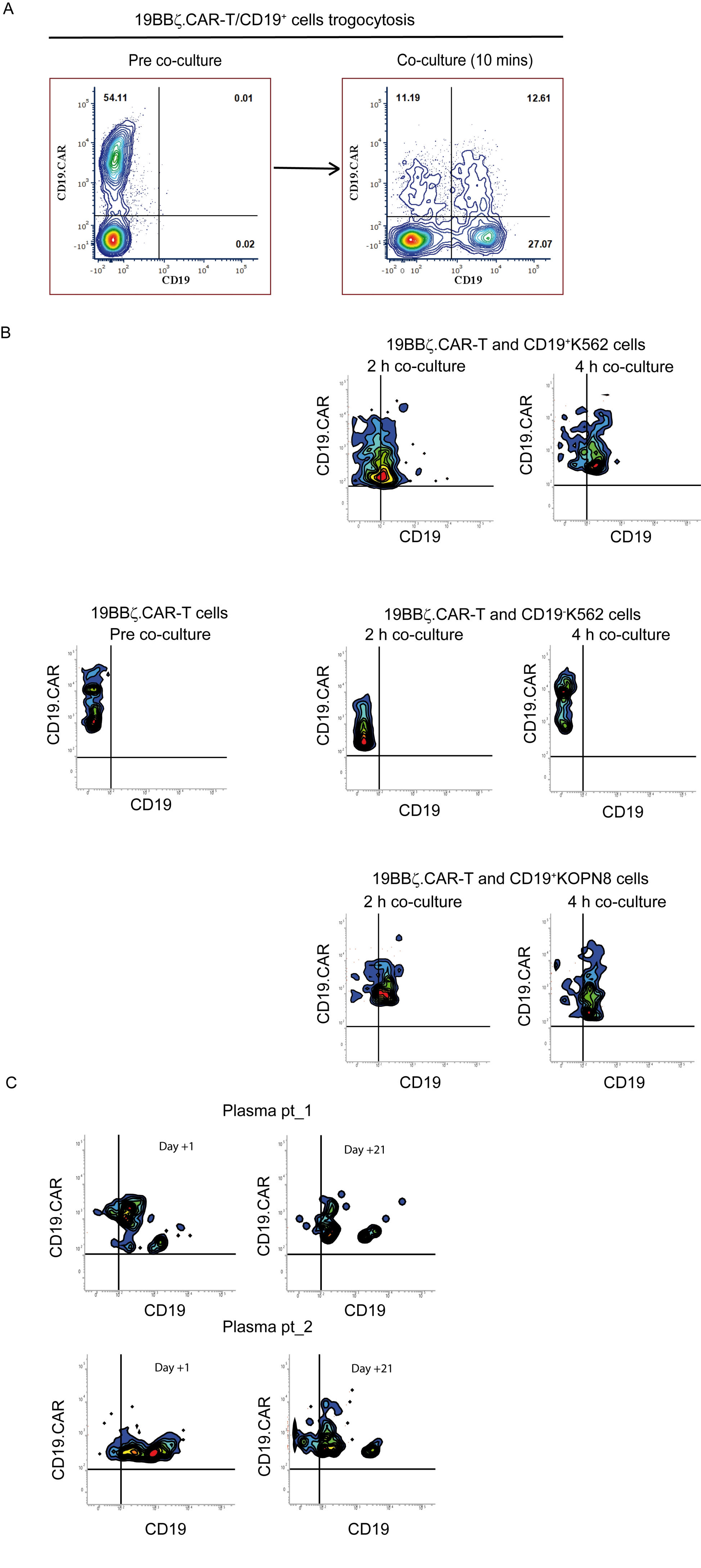

Revised supplementary Figure 8

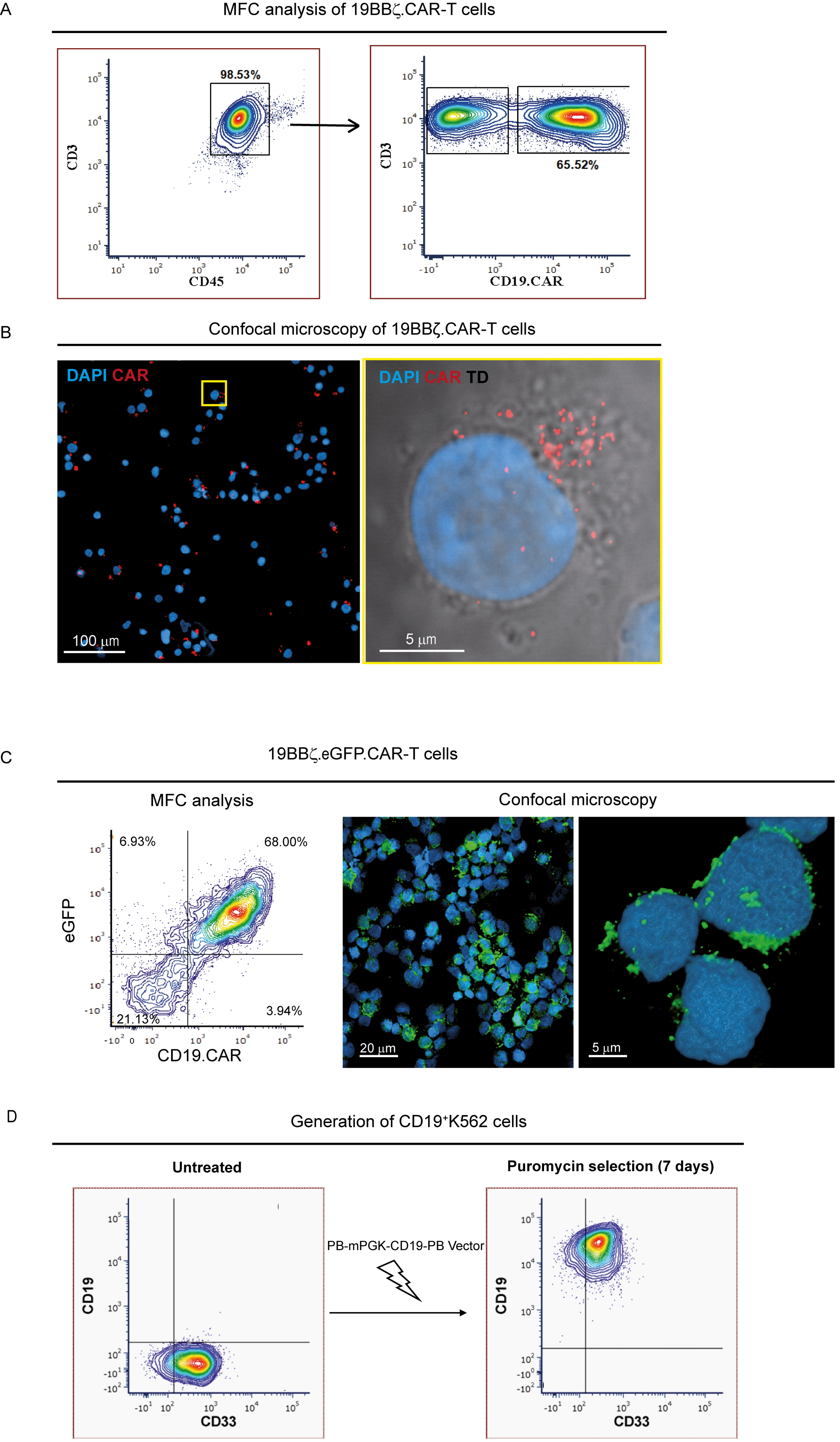

Revised Supplementary Figure 9

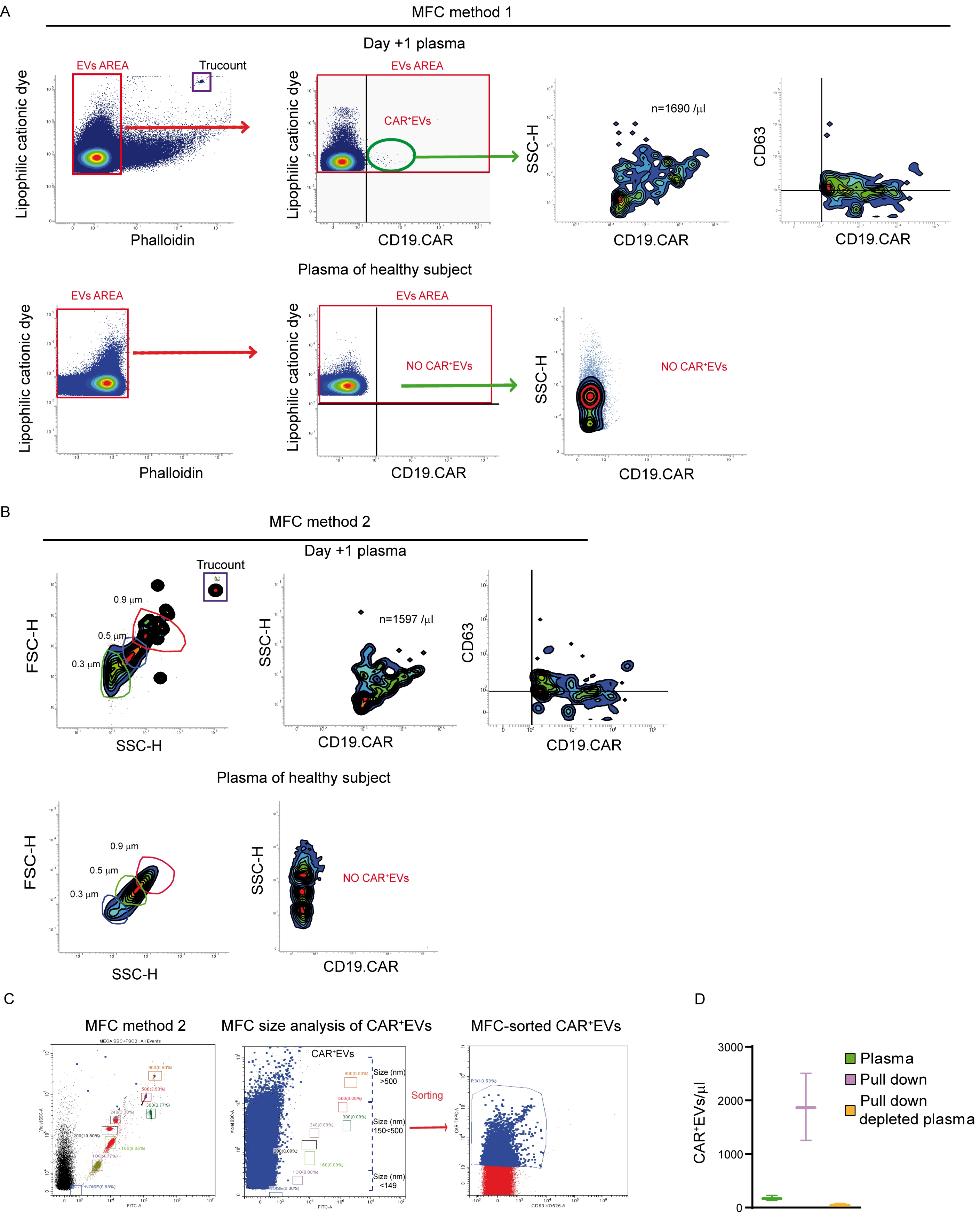

Revised Supplementary Figure 10

Supplemental Figure 1

(A) Representative pictures of 19BBζ.CAR-T cells co-cultured with CD19$^-$K562 or CD19$^+$KOPN8 cells at different time points. Yellow arrowheads indicate dead cells; **(B)** luciferase assay of 19BBζ.CAR-T cells-induced cell death in CD19$^+$KOPN8 cells after 24 h of co-culture (n=3, mean±SD); **(C)** movie snapshot (see Supplemental Video 2) of 19BBζ.eGFP.CAR-T cells co-cultured with PKH26-stained CD19$^+$KOPN8 cells at different time points; **(D)** MFC analysis of supernatants from 19BBζ.CAR-T or 19BBζ.eGFP.CAR-T cells and CD19$^+$KOPN8 cells co-cultures.

Supplemental Video 1 and Video 2

Time-lapse movie of: **(1)** 19BBζ.CAR-T cells and **(2)** 19BBζ.eGFP.CAR-T cells co-cultured with PKH26-stained CD19$^+$KOPN8 cells.

Supplemental Figure 2

(A) Giemsa staining (yellow arrowheads mark T-cells with high content of cytotoxic granules, scale bar: 20 µm) and multicolor staining of 19BBζ.CAR-T cells: DAPI (blue), CD19.CAR (red), CD63 (green) and PRF1 (purple), TD (transmitted light differential interference contrast image, scale bar: 5 µm); white arrowheads mark CD19.CAR non-transduced cells; 3D rendering of 19BBζ.CAR-T cells and co-localization insets are shown, (n=3); **(B)** confocal microscopy of DAPI (Blue), CD19.CAR (red) and PRF1 (purple) staining of a MFC-sorted a 19BBζ.CAR-T cell, (n=10).

Supplemental Figure 3

(A) ExoView platform analysis of plasma CAR$^+$EVs (n=14), showing CD63/CD9/CAR antigens colocalization on anti-CD9/CD63/CD81 Abs immuno-captured EVs (see also Figure 5A, B); note that "low" refers to the detection of the antigen (e.g. CD63) only by the immunocapture Ab, but not by the the fluorochrome-conjugated Ab specific for the same antigen, "high" refers to the detection of the antigen (e.g. CD63) both by the immuno-capture Ab and by the fluorochrome-conjugated Ab

specific for the same antigen. The phenomenon can be explained by the low amounts of a specific antigen (e.g. CD63) on the EVs surface, coupled with the clustering of the antigen on EVs (as also described in McNamara et al., 2022, see Figure B, Figure 5H) that is likely to limit the antigen availability for immuno-detection; **(B)** ExoView platform analysis of size-exclusion chromatography (SEC)-purified EVs from CD19.CAR-T patient's plasma (n=4), pie chart represent the tetraspanin profile of CAR$^+$EVs; **(C)** ExoView platform analysis of EVs from BL (n=3), pie chart represent the tetraspanin profile of CAR$^+$EVs; **(D)** ExoView platform analysis of SEC-purified EVs from BL (n=2), pie-chart represents the % of PRF1$^+$CAR$^+$EVs over total CAR$^+$EVs; **(E)** representative MFC analysis of PRF1$^+$ and CD19.CAR proteins in EVs from CD19.CAR-T patient's plasma; **(F)** MFC analysis of PRF1$^+$CAR$^+$EVs levels in CD19.CAR-T patients plasma (n=6) and BL (n=3); **(G)** ExoView platform analysis of CD107a, WGA and CAR on plasma EVs (n=4); **(H)** MFC analysis of CD107a, WGA, CAR protein on plasma EVs (n=4); **(I)** representative MFC analysis of CD45 protein on plasma CAR$^+$EVs. Data are presented as boxes and whiskers; boxes show median and IQR, whiskers represent minimum and maximum values.

Supplemental Figure 4

(A) Nanosight Tracking analysis of (NTA) MFC-sorted (n=2) and anti-CD19.CAR Ab pull down separated CAR$^+$EVs (n=2) from plasma and BL, respectively; **(B)** NTA assayed CAR$^+$EVs: size range classes distribution is shown; MFC analysis of MFC-sorted plasma CAR$^+$EVs for **(C)** ZAP70, CD63, CD8β proteins (mean±SD), **(D)** FAS-L and CD151 proteins, **(E)** CD107a and CD151 proteins; **(F)** MFC analysis of MFC-sorted CAR$^+$EVs from BL, assed for ZAP-70, CD63 and CD8β proteins.

Supplemental Figure 5

MFC analysis of CD4 and CD8 proteins on CAR$^+$EVs in **(A)** whole plasma (n=6), **(B)** anti-CD19.CAR Ab pull down (n=3), **(C)** BL (n=2); data are shown as mean±SD.

Supplemental Figure 6

(A) Heat map of Pearson's correlation coefficients for hour+1 and day+1 CAR$^+$EVs with pre-lymphodepletion (_PLD) and day+1 biochemical parameters, day+7 CAR_DNA copies and CAR$^+$T cells, and peak levels of CAR$^+$CD8$^+$T$_{EM}$ cells; **(B)** heat map of Spearman's correlation coefficients of ICANS grade with hour+1 and day+1 CAR$^+$EVs, day+1 biochemical parameters, day+7 CAR$^+$T cells and day+7 CAR_DNA copies; **(C** and **D)** NTA of plasma total EVs in NO ICANS (n=26) vs ICANS (n=14) patients at hour +1 and day +1, respectively; MW test. Data are presented as boxes and whiskers; boxes show median and IQR, whiskers represent minimum and maximum values.

Supplemental Figure 7

(A) MFC analysis of hour+1 plasma CAR$^+$EVs in NO CRS (n=8) vs CRS (n=79); **(B)** day+1 plasma CAR$^+$EVs in NO CRS (n=8) vs CRS (n=77); **(C)** hour+1 plasma CAR$^+$EVs in G0-G1 CRS (n=61) vs G≥2 CRS (n=26) and **(D)** day+1 plasma CAR$^+$EVs in G0-G1 CRS (n=58) vs G≥2 CRS (n=27), MW test. Data are presented as boxes and whiskers; boxes show median and IQR, whiskers represent minimum and maximum values.

Supplemental Figure 8

(A) MFC analysis of 19BBζ.CAR-T cells and CD19$^+$KOPN8 cells co-cultures (10 minutes): the presence of CD19$^+$CAR$^+$ cells is highlighted; Representative MFC analysis of CD19$^+$CAR$^+$EVs in the supernatant of: **(B)** 19BBζ.CAR-T cells co-cultured with CD19$^-$K562, or CD19$^+$K562, or CD19$^+$KOPN8 cells at different time points; **(C)** representative MFC analysis of CD19$^+$CAR$^+$EVs in CAR-T patient's plasma at day+1 and day+21, (n=2).

Supplemental Figure 9

(A) MFC analysis of healthy donors T-cells before and after transduction with 19BBζ.CAR expression vector; **(B)** confocal microscopy analysis and 3D rendering of 19BBζ.CAR-T cells: DAPI (blue) and CD19.CAR (Red), (n=9); **(C)** MFC and confocal microscopy analysis of T-cells transduced with 19BBζ.eGFP.CAR expression vector, (n=3); **(D)** MFC analysis of K562 cells transduced with CD19 protein-expressing Piggy Back vector, before and after Puromycin selection.

Supplemental Figure 10

CAR$^+$EVs MFC analysis: gating strategy **(A)** method 1 and **(B)** method 2 performed on a healthy subject plasma and on a patient plasma one day after CD19.CAR infusion: number of CAR$^+$EV is reported, co-expression of the CD63 exosomal marker on CAR$^+$EVs is shown; **(C)** MFC-sorting strategy of plasma CAR$^+$EVs by method 2: Megamix size distribution assessed by violet-laser side scatter (SSC) vs FITC, CAR$^+$EVs size distribution, and presence of CD63 exosomal marker on MFC-sorted CAR$^+$EVs; **(D)** MFC analysis of CAR$^+$EVs concentration in: CAR-T patient's plasma (n=3), anti-CD19.CAR Ab plasma pull down (n=3), and anti-CD19.CAR Ab pull down-depleted plasma (n=3); Data are presented as boxes and whiskers; boxes show median and IQR, whiskers represent minimum and maximum values.

Storci and De Felice_Supplementary Methods

Generation of 19BBζ.CAR-T and 19BBζ.eGFP.CAR-T cells

Replication defective, third-generation lentiviral vectors, namely 19BBζ.CAR-T (pLV[CAR]-EF1A>CD8-leader/CD19-scFV/CD8-hinge/CD8-TM/4-1BB-CD3zeta_VB220923-1237mwu) and 19BBζ.CAR-T eGFP tagged (19BBζ.eGFP.CAR-T) (pLV[CAR]-EF1A>CD8-leader/CD19-scFV/CD8-hinge/CD8-TM/4-1BB-CD3zeta:Linker:EGFP_VB230125-1128xqf) vectors were purchased from Vector builder (Vector builder, Chicago, USA), and were produced using HEK293T cells (ATCC, American Type Culture Collection, Virginia, USA,

RRID:CVCL_HA71(SciCrunch | Research Resource Resolver). Briefly, 0.6×10^6 cells were plated in a 10 cm culture dish (Corning, New York, USA) in DMEM, high glucose (Corning). After 72 hours, cells were transfected using Lentiviral Packaging Kits (OriGene, Rockville, Maryland, USA, TR30037 Lenti-vpak packaging kit - packaging plasmids and transfection reagent) together with 7 µg of 19BBζ.CAR-T or 19BBζ.eGFP.CAR-T lentiviral vectors (Vector builder). Lipofectamine and plasmids DNA were diluted in Opti-MEM media (Gibco by Thermo Fisher Scientific, Whitman, Massachusetts, USA). After 16 hours the medium was changed, and after 48 and 72 hours of lipofection, lentiviral culture medium was collected and concentrated using Lentivirus concentration solution (OriGene). For T-cell engineering, $CD3^+$T cells, derived from seven healthy blood donors, were isolated with CD3 microbeads (Miltenyi Biotec, Bergisch Gladbach, Germany). The isolated $CD3^+$T cells were cultured in TexMACS medium (Miltenyi Biotec) in presence of 10 ng/ml of IL-7 (Cat# 130-095-367, Miltenyi Biotec), IL-15 (Cat# 130-095-760, Miltenyi Biotec) and stimulated for 48 hours with T Cell TransAct (Cat#130-128-758 Miltenyi Biotec). After 48 hours, stimulated T-cells were combined with 19BBζ.CAR-T or 19BBζ.eGFP.CAR-T lentiviral vector at MOI (multiplicity of infection) 20 and cultured in 24 multi-wells plates (Corning) coated with retronectin (Takara Bio, Kusatsu, Japan). After 3 days, CD19.CAR protein expression was assessed by human recombinant APC-conjugated CD19 CAR FMC63 Idiotype antibody (Ab, Cat# 130-127-343 Miltenyi Biotec) or by confocal microscopy and by direct eGFP fluorescence detection, using FACSCanto II cytometer (BD Biosciences, San Jose, California, USA) (Supplementary Figure 9A-C).

Cell lines culture condition and engineering of KOPN8 and K562

HEK293T (Takara Bio, St Germain en Laye, France) were cultured in DMEM high glucose (Corning) supplemented with 10% FBS (Gibco by Thermo Fisher Scientific), 2mM of L-glutamine and 2mM of penicillin-streptomycin (Gibco by Thermo Fisher Scientific). KOPN8 (DSMZ, German Collection of Microorganisms and Cell Cultures GmbH, Braunschweig Germany, RRID:

CVCL_1866 SciCrunch | Research Resource Resolver) and K562 (DSMZ, German Collection of Microorganisms and Cell Cultures GmbH, RRID: CVCL_0004 SciCrunch | Research Resource Resolver) were cultured in RPMI (Corning) supplemented with 10% FBS, 2 mM penicillin-streptomycin and 2 mM of L-glutamine. KOPN8 cells were engineered to express firefly luciferase. Briefly, cells were transduced with Lenti-luciferase-P2A-Neo (Lenti-luciferase-P2A-Neo was a gift from Christopher Vakoc, Addgene plasmid#105621; http://n2t.net/addgene:105621; RRID: Addgene_105621, Addgene, Watertown, Maryland, USA) lentiviral vector and selected with G418 (Thermo Fisher Scientific) for 96 hours. K562 cells were engineered to express CD19 receptor using PiggyBac vector encoding for CD19 cDNA and a puromycin resistance cassette under control of mouse phosphoglycerate kinase promoter (PGK) (Vector builder, pPB[Exp]-mPGK>hCD19[NM_001178098.2](ns):T2A:Puro VB220309-1186msb). Briefly, 1 week after thawing, 0.8×10^6 K562 cells were electroporated with Lonza 2D nucleofector system (Lonza, Basel, Switzerland) in presence of 1 μg of plasmid encoding Transposase (Vector builder) and 3 μg of PiggyBac CD19 plasmid. 72 hours after electroporation, properly transfected cells were selected adding 3 μg of Puromycin (Sigma-Aldrich by Merck Millipore, Darmstadt, Germany) to the medium for 72 hours. After selection, the presence of CD19 receptor was assessed by MFC, using the anti-human mouse monoclonal Ab CD19 PE-Cy7, clone SJ25C1 (BD Biosciences Cat# 341113, RRID: AB_2868769) in a FACSCanto II cytometer (BD Biosciences, Supplementary Figure 9D).

Induced pluripotent stem cells (iPSCs) culture and differentiation

Previously reprogrammed and characterized healthy donor iPSCs (Supplementary Reference 1) were maintained in feeder free conditions and cultured in six well plates (Corning) coated with Vitronectin (Thermo Fisher Scientific) in Essential 8 medium (Thermo Fisher Scientific) as previously described (Supplementary Reference 2). Mature midbrain neurons were obtained by first differentiating iPSCs to the neural precursor cell (NPC) stage (Supplementary Reference 3) and subsequently stimulating the differentiation towards the ventral central nervous system as previously

described (Supplementary Reference 4). Mature NPCs and neurons were seeded on Matrigel-coated plates for functional assays (see below) and imaging by confocal microscopy (see below).

Cytotoxicity and MTT assays

To test the killing capacity of 19BBζ.CAR-T cells, they were co-cultured at a 1:1 ratio with CD19-engineered KOPN8 or K562 cells. The killing rate was measured by flow cytometry after 24 hours as percentage of residual $CD19^{+}$ living cells. 19BBζ.CAR-T cell-induced cell death in KOPN8 engineered to express luciferase was measured by luciferase assay: after 24 hours, the amount of residual KOPN8 cells was evaluated through the Steady-Glo Luciferase Assay System (Cat# E2510, Promega, Madison, Wisconsin, USA) and the luminescence plate reader (Tecan, Männedorf, Switzerland). Metabolic activity of neurons administered or not with CAR^{+}EVs were evaluated with MTT cell proliferation assay (Roche Life science, Milan, Italy).

CAR_DNA molecular tracking by droplet digital PCR (ddPCR)

DNA was extracted from 200 µl of whole blood using the QIAamp DNA Mini and Blood Mini kit (Cat#51104, Qiagen, Hilden, Germany) and quantified with NanoDrop™ One (Thermo Fisher Scientific). Droplet Digital PCR technology (Bio-Rad, Hercules, California, USA) was performed using the Axi-Cel/Tisa-Cel CD19.CAR-T assay (Expert Design Assay, dEXD88164642, 95 bp amplicon, Bio-Rad), duplexed with RPP30 Copy Number assay (dHsaCP2500350, Bio-Rad). A digestion with HaeIII restriction enzyme (Cat#ER0151, Thermo Fisher Scientific) was performed on DNA from whole blood by directly adding the restriction enzyme mix to the ddPCR reaction. Probe-based Droplet Digital PCR mix was set adding 10 µl of 2X QX200 ddPCR Supermix for Probes (no dUTP) (Cat#1863023, Bio-Rad), 1 µl of 20 X Axi-Cel/Tisa-Cel CD19 CAR-T assay (labeled with a 5' 6-FAM fluorophore and a 3' lowa Black®FQ dark quencher), 1 µl of 20X RPP30 Copy Number Determination Assay (labeled with a 5'HEX fluorophore and a 3' lowa Black®FQ dark

quencher), 1 µl of HaeIII (5 U/µl) restriction enzyme mix (Thermo Fisher Scientific) and a variable volume of DNA sample and/or nuclease-free water to reach a total volume of 20 µl. A fixed volume of DNA (2 µl) was used as input in the ddPCR reaction, when the DNA amount was in the range of 20-80 ng. Thermal cycling conditions were: 95°C for 10 min, 40 cycles of 94°C for 30 s and 60°C for 1 min, followed by an enzyme deactivation step at 98°C for 10 min and a 4°C infinite hold. Droplet selection was performed individually for each well using QuantaSoft™ Software version 1.7 (Cat #1864011, Bio-Rad). Absolute copies of CAR per ml of blood were calculated by multiplying the ddPCR concentration (copies/µl) for the dilution factor and then adjusting for 1 ml of blood.

miR-1246 plasma levels quantification by ddPCR

Total RNA, including miRNA, was purified from 200 µl of plasma from 20 patients collected at day +5, using Maxwell RSC miRNA plasma and serum kit and Maxwell RSC instrument according to manufacturer's instructions (Cat#AS1680, Promega). A volume of 3 µl of RNA was converted into cDNA by using the miRCURY LNA RT kit (Cat#339340, Qiagen). Evagreen-based Droplet Digital PCR was performed to assess miR-1246 levels. Briefly, 20 µl of PCR reaction containing 10 µl of 2X EvaGreen Supermix (Cat#186-4033, Bio-Rad), 1 µl of miRCURY LNA miRNA assay (Cat#339306, GeneGlobe ID - YP00205630, Qiagen), 1 µl of nuclease-free and 8 µl of diluted cDNA (1:50). Thermal cycling conditions were: 95°C for 5 min, 40 cycles of 95°C for 30 s and 58°C for 1 min, and three final steps at 4°C for 5 min, 90°C for 5 min and a 4°C infinite hold. Data analysis was performed using QuantaSoft™ Software version 1.7 (Cat #1864011, Bio-Rad) and the absolute level of circulating miR-1246 was obtained by multiplying the ddPCR concentration (copies/µl) for the dilution factor.

Multiparametric flow cytometry (MFC): CAR^{+}T cells tracking and sorting

CAR-T cells tracking was performed on fresh whole blood at all available time points. Cells were stained with the APC-conjugated-CD19 CAR FMC63 Idiotype Ab (Miltenyi Biotec Cat#130-127-

343) following the manufacturer's instructions. Then, cells were labeled with the following set of fluorochrome-conjugated anti-human mAbs: CD3 FITC, clone SK7 (BD Biosciences, Cat# 340851, RRID:AB_400139), CD8 PE-Cy7, clone HIT8A (BD Biosciences Cat# 566858, RRID:AB_2869912), CD45 V500, clone HI30 (BD Biosciences Cat# 560777, RRID:AB_1937324), CD45RA PerCP-Cy5.5, clone HI100 (BD Biosciences Cat# 563429, RRID:AB_2738199), CD62L BV421, clone DREG-56 (BD Biosciences Cat# 563862, RRID:AB_2738455). MFC analysis was performed using a 3-laser FacsCanto II cytometer (BD Biosciences). A minimum of 50,000 $CD45^{+}$ lymphocytes were recorded for each analysis. Flow cytometry data were analyzed with DiVa 6.1.1 software and FCS Express 7 Reader. Appropriate isotype controls were included for each sample. Before sorting of 19BBζ.CAR-T cells, the cells were counted and stained with recombinant human CD3-FITC clone REA613 (Miltenyi Biotec Cat# 130-113-697, RRID: AB_2726238), CD19 CAR FMC63 Idiotype Ab-APC clone REA1297 (Miltenyi Biotec Cat#130-127-343) and CD45 VioGreen, clone REA747 (Miltenyi Biotec Cat# 130-122-311, RRID:AB_2801892). Then, cells were washed, resuspended in TexMACS medium (Miltenyi Biotec Cat# 130-097-196) and transferred into the MACSQuant Tyto HS Cartridge (Miltenyi Biotec). $CD3^{+}CAR^{+}$T cells were sorted on MACSQuant Tyto (Miltenyi Biotec). Before and after cell sorting, cell purity was analyzed by MACSQuant16 using MACSQuantify software (Miltenyi Biotec).

SmartSEC columns EVs purification

Size-exclusion chromatography was applied to samples destined for cytofluorimetric and ExoView analysis. Following differential ultracentrifugation, EVs pellets were resuspended in 100 μl of PBS (filtered with a 0.1 μm filter) or applied to SmartSEC columns separation (SSEC100A-1, System Biosciences, Palo Alto, CA, USA). The samples were then processed as recommended by the manufacturer's protocol.

EVs purification by differential ultracentrifugation

Briefly, 1 ml of CAR-T patient-derived plasma was serially centrifuged, as follows: 1500×g for 15', 2500×g for 15', 20.000×g for 1h, and 100.000×g for 3h (Beckman Coulter). EVs pellet was immediately resuspended in sterile 0.1 µm-filtered PBS and stored at -80°C.

CAR$^+$EVs pull-down

CAR$^+$EVs were isolated from plasma using an ad-hoc immunoaffinity EVs capture system (Hansa Biomed, Tallinn, Estonia). Briefly, 0.4 µm latex immunobeads covalently coupled with CD19 CAR FMC63 idiotype Ab clone REA1297 (Cat# 130_127_983 Miltenyi Biotec) were mixed over-night with human plasma following manufacturer instructions. CAR$^+$EVs were detached from immunobeads following manufacturer instructions, counted by MFC (Supplementary Figure 10D) and sized by Nanosight Tracking Analysis (NTA) as described below.

EVs Nanosight Tracking Analysis (NTA)

The EVs size and concentration were determined by NTA using the NanoSight RS300 system (Malvern Panalytical, Malvern, United Kingdom). Each sample was diluted (1:100) in PBS (filtered with a 0.1 µm filter). For each sample, a syringe pump flow of 30 was applied. Three videos of 60s each were recorded and analyzed, calculating an average number of EV size and concentration (particles/ml). All samples were characterized with NTA 3.2 analytical software.

ExoView platform analysis

Whole CAR-T patient plasma EVs, infusion BL EVs and SmartSEC columns isolated EVs, were analyzed by ExoView platform™ (NanoView Biosciences, Maryland, USA), following the manufacturer's protocol. Abs used for EVs detection were: recombinant human PE Conjugated CD19 CAR FMC63 Ab Idiotype (Miltenyi Biotec), fluorochrome-conjugated anti human mAbs: CD107a APC-H7, clone H4A3 (BD Biosciences Cat# 561343, RRID: AB_10644020), Perforin Alexa-647, clone dg9 (BioLegend Cat# 308110, RRID:AB_493254), CD9 Alexa-488, CD81 Alexa-555 and

CD63 Alexa-647 were provided by Nanoview Biosciences. Recombinant Human CD19 Fc Chimera Alexa Fluor® 647 Protein (Cat#AFR9269, Biotechne, Minnesota, USA). Alexa-488 Wheat Germ agglutinin (WGA) was purchased from Invitrogen (Thermo Fisher Scientific). The analysis was performed according to the manufacturer's instructions, using the ExoView™R200 reader endowed with ExoView Scanner software (v 3.0).

Protein Ella Cytokine assay

Plasma level of Interleukins (e.g. IL-15, GDF15), chemokines (e.g. CX3CL1, CXCL9) and neurofilaments (NFL) were assessed by high performance multi analyte microfluidic system Protein ELLA (Biotechne).

dSTORM analysis of CAR^{+}EVs with Python script.

Single-EV segmentation was performed using DBSCAN in NEO_analysis (Abbelight) with a search radius of 150 nm. Biomarker positivity for each EV was performed with custom Python script. Briefly, the script (available in the SI) takes as inputs the coordinate table where each localization has been assigned to a cluster ("CoordTable_SAFE360_MULTIPLEXING_demixed_clusters.csv") and the list of cluster properties ("ClustersInfos_DBSCAN_CH#1.csv"), both files are output by NEO_analysis clustering, the script then counts the number of localizations of each channel (AF647 and CF680) for each single cluster. If in a single cluster the number of localizations of either AF647 or CF680 is higher or equal than 10, then the cluster will be labelled with either the "AF647-only" or "the CF680-only" label, if the localizations of both AF647 and CF680 are both higher or equal than 10, then the cluster will be labelled as "Double", if neither the AF647 nor the CF680 localizations will pass this threshold, the cluster will be labelled as "None" and considered as nonspecific background. The threshold of 10 localizations for biomarker positivity was chosen as the value which eliminates >85% of objects seen in the negative control samples (no plasma). The script outputs

updated coordinate table file ("CoordTable_SAFE360_MULTIPLEXING_demixed_clusters_cluster-color.csv”) and cluster infos file (“ClustersInfos_DBSCAN_CH#1_cluster-color.csv”), where the biomarker positivity information has been assigned to each cluster. Doubly positive clusters are then exported using the data filtering function in excel to generate coordinate tables featuring exclusively doubly positive clusters. .tiff images from those coordinate tables are then generated using the NEO_analysis “export to .tiff” function.

Python code for STORM CAR-EV analysis

```
import math
import pandas as pd
import numpy as np
import time
path = "F:/Demos/Bologna_2023/CAR-EVs/1_plasma-don/" #change for analyzing different folders
file = "CoordTable_SAFE360_MULTIPLEXING_demixed_clusters.csv"
file_noext = "CoordTable_SAFE360_MULTIPLEXING_demixed_clusters"
cluster_file = "ClustersInfos_DBSCAN_CH#1.csv"
cluster_file_noext = "ClustersInfos_DBSCAN_CH#1"
def load_coordinate_table(path, file):
    path_file = path+file
    coordinate_table = pd.read_csv(path_file)
    if not "DBSCAN_cluster_id" in coordinate_table.columns:
        print("Requires the *_clusters.csv exported from Neo Analysis v39")
        quit()
    else:
        return(coordinate_table)
```

```
def load_cluster_infos(path, cluster_file):
    path_cluster_file = path + cluster_file
    cluster_infos = pd.read_csv(path_cluster_file,
                                skiprows= 2,
                                low_memory = False)
    return(cluster_infos)
def assign_cluster_color(cluster):
    min_locs = (10,10)
      try:
        AF647_locs = cluster['Channel'].value_counts()['AF647']
    except KeyError:
        AF647_locs = 0

    try:
        CF680_locs = cluster['Channel'].value_counts()['CF680']
    except KeyError:
        CF680_locs = 0
            if AF647_locs < min_locs[0] and CF680_locs < min_locs[1]:
        cluster_color = "None"
        elif AF647_locs < min_locs[0] and CF680_locs >= min_locs[1]:
        cluster_color = "CF680-only"
    elif AF647_locs >= min_locs[0] and CF680_locs < min_locs[1]:
        cluster_color = "AF647-only"
    else:
        cluster_color = "Double"
    return(cluster_color)
```

```
coordinate_table = load_coordinate_table(path, file)
cluster_infos = load_cluster_infos(path, cluster_file)

cluster_ids = np.unique(np.array(coordinate_table["DBSCAN_cluster_id"], dtype=int))
numClusters = len(cluster_ids)

start = time.time()


for c_id in cluster_ids:
    if (math.floor(c_id) % 1000 == 0):
        print("-> Analyzing cluster {:,} of {:,}".format(c_id,numClusters))
    cluster = coordinate_table.loc[coordinate_table["DBSCAN_cluster_id"] == c_id]
    cluster_color = assign_cluster_color(cluster)
    cluster_infos.loc[cluster_infos["ID_CLUSTER"] == c_id, "cluster_color"] = cluster_color
    coordinate_table.loc[coordinate_table["DBSCAN_cluster_id"] == c_id, "cluster_color"] =
cluster_color
coordinate_table.to_csv(path+file_noext+"_cluster-color.csv")
cluster_infos.to_csv(path+cluster_file_noext+"_cluster-color.csv")
end = time.time()
print("{:,} seconds elapsed".format(end - start))
```

Supplementary References

1. Messelodi D, et al. iPSC-Derived Gaucher Macrophages Display Growth Impairment and Activation of Inflammation-Related Cell Death. *Cells*. 2021;10(11):2822.

2. Bertuccio SN, et al. The Pediatric Acute Leukemia Fusion Oncogene ETO2-GLIS2 Increases Self-Renewal and Alters Differentiation in a Human Induced Pluripotent Stem Cells-Derived Model. *Hemasphere*. 2020;4(1):e319.
3. Reinhardt P, et al. Derivation and expansion using only small molecules of human neural progenitors for neurodegenerative disease modeling *PLoS One*. 2013;8(3):e59252.
4. Messelodi D, et al. Neuronopathic Gaucher disease models reveal defects in cell growth promoted by Hippo pathway activation. *Commun Biol*. 2023;6(1):431.

Supplementary Table 1 Patient's characteristics

**Baseline characteristics**		**Total (n=100)**	**NO CRS (n=10)**	**CRS (n=90)**	**p value**
Age, median (range)		60 years (19-76)	63.5 years (27-70)	59 years (19-76)	0.196
Sex, n (%)	Female	33 (33)	2 (20)	31 (34)	0.571
	Male	67 (67)	8 (80)	59 (66)	
ECOG, n (%)	0	85 (85)	10 (100)	75 (83)	0.351
	≥ 1	15 (15)	0 (0)	15 (17)	
BMI, n (%)	< 25	47 (47)	3 (30)	44 (49)	0.423
	≥ 25	53 (53)	7 (70)	46 (51)	
**Disease characteristics**					
Diagnosis, n (%)	DLBCL	44 (44)	4 (40)	40 (44)	0.326
	t-DLBCL	23 (23)	5 (50)	18 (20)	
	PMBCL	12 (12)	0 (0)	12 (13)	
	HGBCL	6 (6)	0 (0)	6 (7)	
	GZL	1 (1)	0 (0)	1 (1)	
	MCL	14 (14)	1 (10)	13 (15)	
Disease status, n (%)	PD	62 (62)	5 (50)	57 (63)	0.631

Baseline characteristics		Total (n=100)	NO CRS (n=10)	CRS (n=90)	p value
	NO PD (CR/PR/SD)	38 (38)	5 (50)	33 (37)	
**Prior therapies**					
Previous therapies, median (range)		3 (1-11)	3 (2-11)	3 (1-7)	0.073
Previous ASCT, n (%)	Yes	31 (31)	5 (50)	26 (29)	0.313
	No	69 (69)	5 (50)	64 (71)	
Bridging therapy, n (%) *		81 (81)	7 (70)	74 (82)	0.610
	Chemo-based	25 (25)	5 (50)	20 (22)	0.124
	Steroids	16 (16)	0 (0)	16 (18)	0.317
	Radiotherapy	12 (12)	0 (0)	12 (13)	0.473
	Immune Checkpoint Inhibitors	9 (9)	0 (0)	9 (10)	0.641
	BTKi	7 (7)	1 (10)	6 (7)	1.000
**CAR-T cell therapy**					
CAR-T cell product, n (%)	Tisa-cel	28 (28)	6 (60)	22 (24)	**0.045**
	Axi/Brexu-cel	72 (72)	4 (40)	68 (76)	
LDH, median (range)		220 (76 - 8,365)	201 (155 - 389)	220.5 (76 - 8365)	0.498
CRP, median (range)		0.425 (0.02 - 22.93)	0.37 (0.10 - 2.29)	0.44 (0.02 - 22.93)	0.667
Ferritin, median (range)		191 (8 - 7,493)	168 (13 - 1,538)	196 (8 - 7,493)	0.913

CRS: Cytokine Release Syndrome; ECOG: Eastern Cooperative Oncology Group performance scale; BMI: body mass index; DLBCL: Diffuse Large B Cell Lymphoma; t-DLBCL: transformed Diffuse Large B Cell Lymphoma; HGBCL: High Grade B Cell Lymphoma; PMBCL: Primary Mediastinal B Cell Lymphoma; MCL: Mantle Cell Lymphoma; GZL: Grey Zone Lymphoma; PD: Progressive Disease; CR: Complete Response; PR: Partial Response; SD: Stable Disease; ASCT: Autologous Stem Cell Transplantation; BTKi: Bruton Tyrosine Kinase inhibitors; LDH: lactate dehydrogenase; CRP: C-reactive protein. *23 patients underwent miscellaneous bridging therapies.
